# NOTCH dependent cooperativity between myeloid lineages promotes Langerhans cell histiocytosis pathology

**DOI:** 10.1126/sciimmunol.add3330

**Published:** 2022-12-16

**Authors:** Egle Kvedaraite, Paul Milne, Ahad Khalilnezhad, Marion Chevrier, Raman Sethi, Hong Kai Lee, Daniel W. Hagey, Tatiana von Bahr Greenwood, Natalia Mouratidou, Martin Jädersten, Nicole Yee Shin Lee, Lara Minnerup, Tan Yingrou, Charles-Antoine Dutertre, Nathan Benac, You Yi Hwang, Josephine Lum, Amos Hong Pheng Loh, Jessica Jansson, Karen Wei Weng Teng, Shabnam Khalilnezhad, Xu Weili, Anastasia Resteu, Tey Hong Liang, Ng Lai Guan, Anis Larbi, Shanshan Wu Howland, Henrik Arnell, Samir EL Andaloussi, Jorge Braier, Georgios Rassidakis, Laura Galluzzo, Andrzej Dzionek, Jan-Inge Henter, Jinmiao Chen, Matthew Collin, Florent Ginhoux

**Affiliations:** 1 Childhood Cancer Research Unit, Department of Women’s and Children’s Health, Karolinska Institutet, Stockholm, Sweden; 2 Center for Infectious Medicine, Department of Medicine Huddinge, Karolinska Institutet, Karolinska University Hospital, Stockholm, Sweden; 3 Department of Clinical Pathology, Karolinska University Laboratory, Stockholm, Sweden; 4 Translational and Clinical Research Institute, Newcastle University, Newcastle-upon-Tyne, UK; 5 Northern Centre for Cancer Care, Newcastle-upon-Tyne Hospitals NHS Foundation Trust, Newcastle-upon-Tyne, UK; 6 Singapore Immunology Network (SIgN), Agency for Science, Technology and Research (A*STAR), BIOPOLIS, Singapore, Singapore; 7 Clinical Research Center, Department of Laboratory Medicine, Karolinska Institutet, Stockholm, Sweden; 8 Pediatric Oncology, Astrid Lindgrens Children’s Hospital, Karolinska University Hospital, Stockholm, Sweden; 9 Pediatric Gastroenterology, Hepatology and Nutrition Unit, Astrid Lindgren Children’s Hospital, Karolinska University Hospital, Stockholm, Sweden; 10 Department of Medicine Solna, Karolinska Institutet, Stockholm, Sweden; 11 Department of Hematology, Karolinska University Hospital, Stockholm, Sweden; 12 Center for Hematology and Regenerative Medicine, Department of Medicine Huddinge, Karolinska Institutet, Stockholm, Sweden; 13 Miltenyi Biotec B.V. & Co. KG, Bergisch Gladbach, Germany; 14 National Skin Center, National Healthcare Group, Singapore; 15 INSERM U1015, Gustave Roussy Cancer Campus, Villejuif, France; 16 Université de Bordeaux, Interdisciplinary Institute for Neuroscience, UMR 5297, Bordeaux, France; 17 VIVA-KKH Paediatric Brain and Solid Tumour Programme, KK Women’s and Children’s Hospital, Singapore; 18 National Skin Centre, National Healthcare Group, Singapore; 19 Lee Kong Chian School of Medicine, Nanyang Technological University, Singapore; 20 Yong Loo Lin School of Medicine, National University of Singapore; 21 Hospital Nacional de Pediatría Dr Prof JP Garrahan, Pathology Department, Buenos Aires, Argentina; 22 Immunology Translational Research Program, Yong Loo Lin School of Medicine, Department of Microbiology and Immunology, Narional Unietsoty of Sinapore (NUS); 23 Shanghai Institute of Immunology, Shanghai JiaoTong University School of Medicine, Shanghai, China; 24 Translational Immunology Institute, SingHealth Duke-NUS Academic Medical Centre, Singapore, Singapore

## Abstract

Langerhans cell histiocytosis (LCH) is a potentially fatal neoplasm, characterized by the aberrant differentiation of mononuclear phagocytes, driven by mitogen-activated protein kinase pathway activation. LCH cells may trigger destructive pathology yet remain in precarious state, finely balanced between apoptosis and survival, supported by a unique inflammatory milieu. The interactions that maintain this state are not well-known and may offer targets for intervention. Here, we used single-cell RNA-seq and protein analysis to dissect LCH lesions, assessing LCH cell heterogeneity, and comparing LCH cells with normal MNPs within lesions. We found LCH-discriminatory signatures pointing to senescence and escape from tumor immune surveillance. We also uncovered two major lineages of LCH with DC2- and DC3/Monocyte-like phenotypes and validated them in multiple pathological tissue sites by high-content imaging. Receptor-ligand analyses and lineage tracing *in vitro* revealed Notch dependent cooperativity between DC2 and DC3/monocyte lineages, during expression of the pathognomonic LCH program. Our results present a convergent dual origin model of LCH with MAPK pathway activation occurring prior to fate commitment to DC2 and DC3/Monocyte lineages and Notch-dependent cooperativity between lineages driving the development of LCH cells.

## Introduction

Histiocytic disorders are characterized by an accumulation of pathological histiocytes, a term that describes tissue-resident mononuclear phagocytes (MNP) comprising ontogenetically- and functionally- distinct dendritic cells (DC), monocytes and macrophages (1). Recent advances in the field of human MNP biology have significantly improved our understanding of different DC subsets in humans (2), including the newly discovered DC3, that share some phenotypic markers with monocytes (3–5). Langerhans cell histiocytosis (LCH) is the most common histiocytic disorder, most frequently being diagnosed in young children (6), where it may prove fatal in multisystem high-risk cases (7). The pathologic histiocytes or LCH cells are a component of complex inflammatory lesions (7–9) that may develop in multiple organs, causing tissue damage.

LCH cells are identified by expression of CD1a and CD207, which are also features of normal epidermal Langerhans cells and Langerin-expressing DC (10). Following identification of the oncogenic somatic mutation *BRAF^V600E^* in 60% of LCH lesions (11), it became clear that LCH is an inflammatory myeloid neoplasm. *BRAF^V600E^* or other mutations in the MAPK pathway are detectable in hematopoietic stem cells and myeloid lineages (12–20), suggesting that LCH lesions arise by aberrant differentiation of monocyte or dendritic cell lineages bearing mutation. Both monocytes and myeloid DC can express an LCH-like differentiation program but it is not known whether one or both lineages contribute to LCH lesions or whether the recently-described DC3 subset also has the potential to form LCH (13, 17, 20). Furthermore, while the downstream effects of MAPK activation on myeloid differentiation have been described in animal models of LCH (14, 19, 21), development of the pathognomonic LCH program is not well understood in humans.

Here, we conducted deep profiling of LCH cells from patients, combining single-cell RNA-seq and protein analysis, to assess LCH cell heterogeneity and relationship to their normal counterparts within the MNP system. Our results suggest a convergent dual origin of LCH cells and highlight the strong influence of microenvironment cues on LCH cell phenotype and gene expression profile, identifying Notch-dependent cooperativity between DC2 and DC3/monocytes in gaining the pathognomonic LCH state.

## Results

### Multi-omic deconstruction of LCH cells lesions into myeloid cell components

**Fig. 1:**
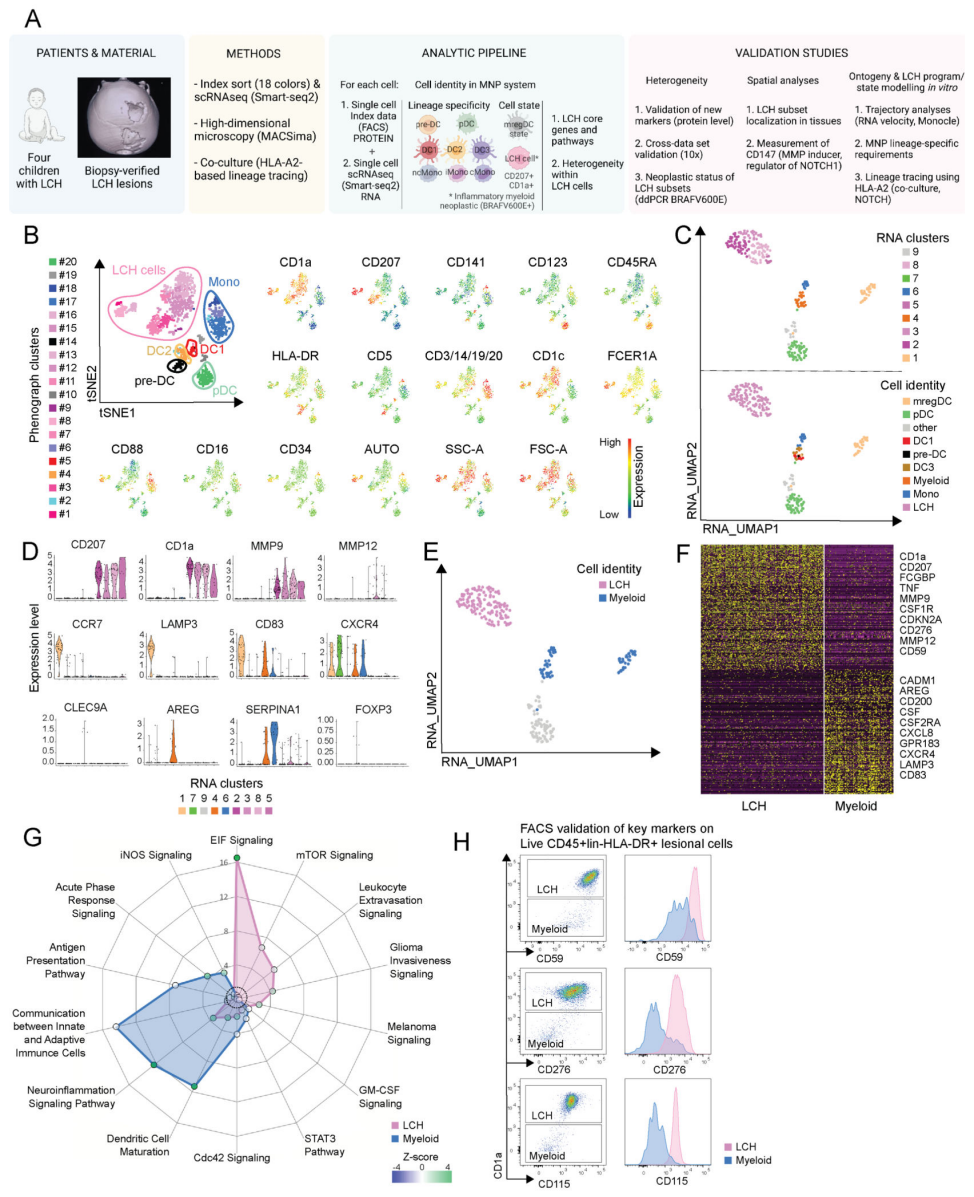
Multi-omic deconstruction of LCH cells lesions into myeloid cell components reveal LCH core signature. (A) Schematic illustration of the study design. (B) Protein expression data from index-sort presented in a tSNE plot with color-coded PhenoGraph clusters; cell identities shown are based on protein level expression of signature markers presented in expression plots. (C) scRNA-seq data presented in a UMAP plot with color-coded PhenoGraph clusters (RNA clusters, upper panel) and further annotated taking protein derived cell identities into account (Cell identity, lower panel); Cell identities: mregDC, pDC, other, DC1, pre-DC, DC3, Myeloid – the remaining cells from the DC cluster 4, Mono, LCH (also see Fig. S1 for gating and backgating). (D) Violin plots showing relative expression level of selected key DEGs in the RNA-data-based-cell-clusters. (E) 2. LCH cells (pink) and other mononuclear myeloid cells (blue) highlighted in a UMAP. (F) Heatmap showing relative expression levels of the top 400 DEGs between LCH cells and other mononuclear myeloid cells; high to low expression indicated as yellow to dark purple. (G) Ingenuity pathway analysis of DEGs in LCH cells (pink) and other mononuclear myeloid cells (blue), displayed as a spider web plot showing log (p value) and Z score for each pathway, calculated using DEGs expressed at significantly higher or lower levels in LCH cells compared to other mononuclear myeloid cells; dashed circle indicates significance level at P<0.05. (H) Gating strategy for extracellular flow cytometry of lesional cells; histograms showing mean fluorescence intensity of expression of the indicated markers on LCH cells (pink) and other mononuclear myeloid cells (blue).

To understand how LCH cells differ from other myeloid cells in LCH lesions at the single-cell level, we index-sorted bone lesion cells from four children with LCH (Table S1, Patients 1-4), enriched for mononuclear myeloid cells and sequenced the resulting populations using the Smart-seq2 pipeline (Fig. 1A and Methods). Next, we subjected the index data of protein expression for each cell to unsupervised clustering, which identified CD1a^+^CD207^+^ LCH cells, other major lesional myeloid subsets, and plasmacytoid DCs (pDC) (Fig. 1B). Dimension reduction analysis and clustering based on RNA expression profile revealed nine PhenoGraph clusters, which we visualized in a UMAP projection (Fig. 1C and Methods). This revealed a close overlap between the RNA data- and protein-based cell identities within the LCH lesions (Fig. 1C), defined using both unsupervised clustering (Fig. 1B) as well as manual gating (Fig. S1A-E). Based on the protein data, we identified seven cell populations in LCH lesions: CD141^+^DC1, cDC2 which comprised CD5^+^DC2 and CD5^-^DC3, CD141^+^CD123^+^pre-DC/AS DC, CD123^+^pDC, CD88^+^CD14^+^ monocytes, and CD1a^+^CD207^+^ LCH cells (Fig. 1B and Fig. S1A-C). Although there was inter-individual variation in expression of extracellular CD207 protein (Fig. 1B and Fig. S1F), this did not bias the distribution of RNA clusters towards any specific patient (Fig. S1G).

When we examined the differentially-expressed genes (DEG) between the RNA data defined clusters, in line with previously described signatures (4, 22, 23), we defined the identities of nine lesion cell populations: clusters 1 and 4 corresponded to DC, cluster 6 to monocytes, cluster 7 to pDC, cluster 9 to contaminating lymphoid cells (annotated as “other”) and clusters 2, 3, 5 and 8 to LCH cells (Fig. S1H). These latter clusters showed a shared gene expression profile including key LCH genes, such as *CD1a* and *CD207* (Fig. 1D). Of note, genes such as *LAMP3*, *CCR7* and *CD83*, that were recently suggested to distinguish different LCH subpopulations in a 10X data set (24) were indeed present in cells from the LCH lesions, but were instead expressed by the population of newly-discovered DCs found in tumors, so called “mature DC enriched in immunoregulatory molecules” (mregDC) (25), and not by LCH cells (Fig. 1D). DCs in the mreg state expressed higher levels of CCR7, LAMP3, and CD83 (cluster 1 in Fig. S1H), and, as expected, corresponded to different DC subsets defined by surface antigen expression (Fig. S1C). In addition, we found that *CLEC9A* was only expressed in the DC1-containing cluster 4, in contrast to a previous study (24), and CXCR4, which was also previously associated with LCH cells (26), was expressed by all clusters of lesion cells, except for LCH cells (Fig. 1 D). Our data instead confirmed that the previously described LCH-cell-associated genes *CD207*, *CD1a*, as well as the matrix metalloproteases *MMP9* and *MMP12*, were expressed by LCH cells (Fig. 1D). In addition, separation between LCH cells and other lesion MNPs was also evident on the regulatory network level, that revealed neoplasm/cancer related regulons specifically active in LCH cells (ERG1, ETV5, ZMIZ1) (Fig. S2). Altogether, this analysis revealed a clear separation between LCH and other lesion MNPs at the RNA and protein level with single-cell resolution. Moreover, we were able to discriminate those previously-proposed LCH-cell-associated expressed genes that were in fact expressed by non-LCH cells within lesions from *bona fide* LCH-cell-specific expressed genes.

### LCH cells express a signature surface marker phenotype and a senescence-associated gene expression profile

To define the core gene signature discriminating LCH cells from non-LCH myeloid cells, we performed detailed DEG analysis between LCH cells (clusters 2, 3, 5, 8 highlighted in pink), and non-LCH myeloid cells (clusters 1, 4, 6 highlighted in blue) (Fig. 1E). As expected, known LCH cell genes such as *CSF1R*, *TNF*, *CD1a*, and *CD207*, were among the top DEG between lesion LCH and non-LCH myeloid cells (Fig. 1F). Pathway analysis confirmed these observations, highlighting innate immune responses, inflammation and cancer signaling as key pathways, as expected of an inflammatory myeloid neoplasm (Fig. 1G). Interestingly, the tumor suppressor *CDKN2A* (p16) was also among the top most DEG, along with other aging-associated genes, such as *FCGBP* (27), pointing to a possible role of senescence mechanisms in these cells. Investigating this further, we detected enriched expression of genes involved in CDC42 and mTOR signaling – both known drivers of cellular senescence – in LCH cells, with the most prominent genes enriching these pathways being *CDKN2A* (p16) and *CCND1* (Cyclin D1). Further aging-related genes including *LMNA* and p21-activating kinase (*PAK1*) were also among the DEG more highly expressed in LCH cells than other myeloid cells within the lesions, alongside genes pointing to a senescence-associated secretory phenotype, such as *TNF* and chemokine like factor *CKLF* (Fig. S1H). To validate the gene expression data, we compared protein level expression of p16, mTOR and p53, which have key roles in cellular senescence, in LCH cells and other lesion MNPs by phosphoflow cytometry (Fig. S1J). We found that all three proteins were expressed at higher levels on LCH cells compared to other myeloid cells in lesions (Fig. S1J), confirming a relative enrichment of the senescence phenotype in LCH cells.

Other features that differed between LCH and non-LCH myeloid cells in the lesion included higher expression of CD115 (CSF1R), CD59 and CD276 which were validated at the protein level (Fig. 1H, Fig. S1K), and were also compared between Langerhans cells and myeloid cells from healthy skin (Fig. S3); and significantly lower levels of expression of genes encoding MHC class I molecules (*HLA-A*, *HLA-B*, *HLA-E*, *HLA-F*; Fig. S1K).

### Two major LCH subpopulations reveals distinct immune phenotype and regulatory network activity

**Fig. 2:**
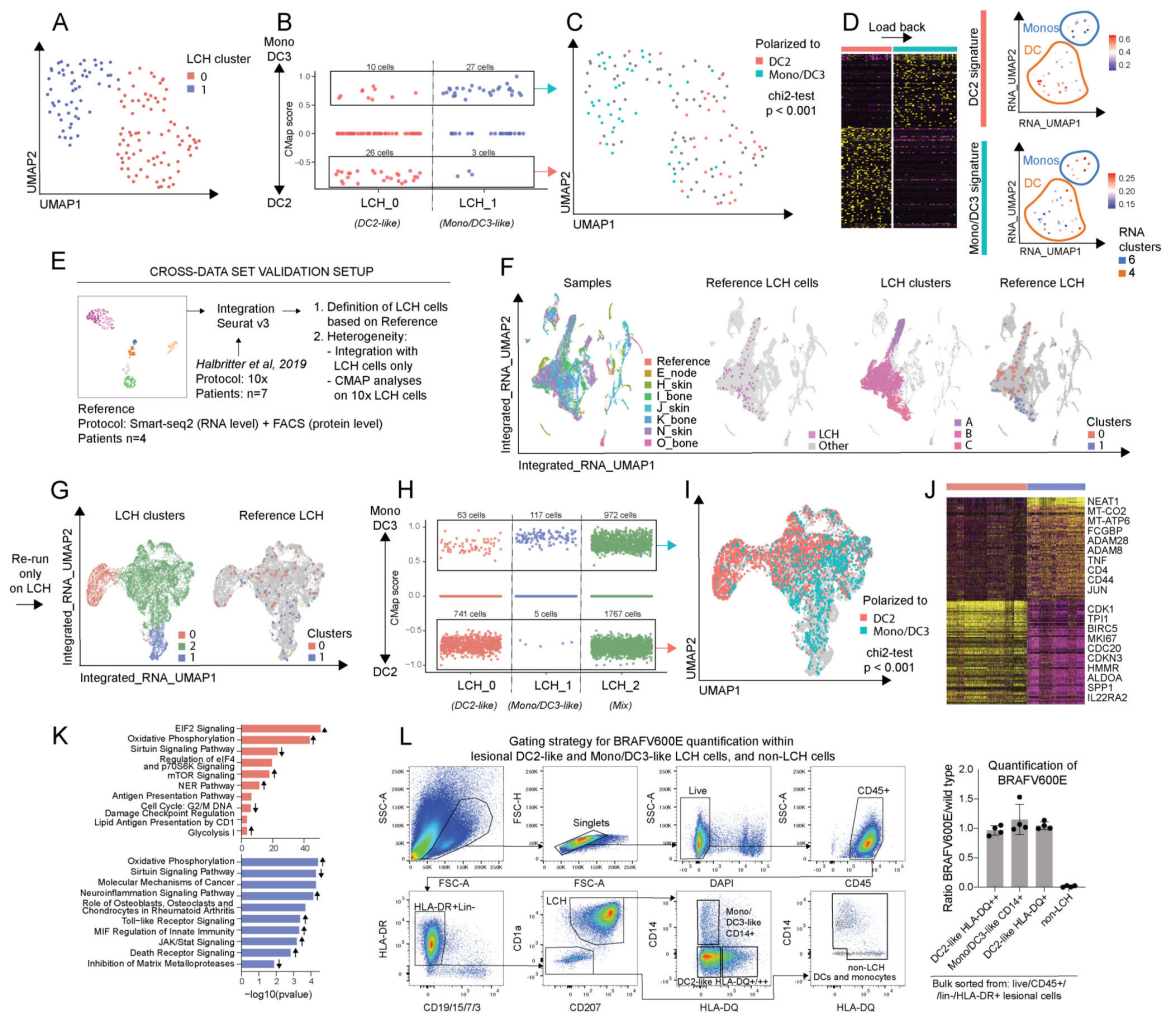
LCH cell heterogeneity, cross-data set validation, and BRAFV600E validation. (A) LCH cells subjected to PhenoGraph clustering (indicated as LCH cluster) based on gene expression data, presented in a UMAP. (B) LCH cells subjected to CMAP analysis using DC2 and DC3/Mono signatures in the two LCH clusters; cells polarized towards DC2 and DC3/Mono (red and light green arrow, respectively) from both clusters boxed separately. (C) Cells polarized towards DC2 (red) and DC3/Mono (light green) from (B) plotted back onto a UMAP from (A). Distribution of DC2-polarized and DC3/Mono-polarized LCH cells in the two LCH clusters assessed using chi2 test. (D) Heatmap showing the relative expression level of the top 200 DEGs between the DC2-polarized and DC3/Mono-polarized LCH cells and mean expression level from the respective signature shown on the RNA-data-based-cell-clusters 4 and 6 from Fig 1C, representing DC and Monos, respectively; high to low expression indicated as yellow to dark purple. (E) Schematic illustration of cross-data set validation pipeline. (F) Integrated 10X and Smart-seq2 data sets presented in a UMAP with annotations for samples from 10X, Smart-seq2 LCH cells (indicated as Reference LCH cells), 10X SNN clusters containing Reference LCH cells (A, B and C, indicated as LCH clusters), and cells from the two LCH clusters 0 and 1 (indicated as Reference LCH). (G) Integrated LCH cells only, from 10X and Smart-seq2 data presented in a UMAP with annotations for 10X SNN clusters (0, 2 and 1, indicated as LCH clusters), and cells from the two Smart-seq2 LCH clusters 0 and 1 (indicated as Reference LCH). (H) LCH cells from 10X data set subjected to CMAP analysis using DC2 and DC3/Mono signatures in clusters 0, 2 and 1; cells polarized to DC2 and DC3/Mono (red and light green arrow, respectively) boxed separately. (I) Cells polarized towards DC2 (red) and DC3/Mono (light green) from (H) plotted back onto a UMAP from (D). Distribution of DC2-poralized and DC3/Mono-polarized LCH cells in the 10X LCH_0 and LCH_1 clusters assessed using chi2 test. (J) Heatmap showing relative expression level of the top 600 DEGs in 10X LCH clusters LCH_0 (red) and LCH_1 (blue); high to low expression indicated as yellow to dark purple. (K) Ingenuity pathway analysis of DEGs between the two LCH clusters (red and blue), significant Z score is indicated by arrow direction for genes expressed at a higher (up) or lower (down) level. (L) Gating strategy for LCH cells and other lesional mononuclear myeloid cells (left) and ratio of BRAFV600E to wild type cells detected by ddPCR in bulk sorted lesional cells from four patients: Mono/DC3-like (CD14^+^ LCH cells), DC2-like (CD14^-^HLA-DQ^+^ and CD14^-^HLA-DQ^++^ LCH cells), and non-LCH cells (HLA-DR^+^Lin^-^CD1a^-^CD207^-^) (right, see also Fig. S7 for ddPCR controls).

We next assessed the level of heterogeneity within LCH cells by extracting and reclustering only these cells. This revealed two major clusters: cluster LCH_0 and LCH_1 (Fig. 2A), with uniform distribution within each patient (Fig. S4A). We next looked at the DEG and differentially-enriched pathways between the two clusters. We identified *CDC20*, an essential cell division regulator, and DC-specific MHC class II molecule *HLA-DPA1* as highly expressed in cluster LCH_0; whereas *LYZ*, mitochondrial genes and *SOD2* were expressed highly in cluster LCH_1 (Fig. S4B). Pathway analysis revealed evidence of senescence mechanisms in both clusters, but with distinct patterns: mTOR, CDC42, p7056K signaling and glycolysis predominated in cluster LCH_0; while we saw high expression of genes involved in oxidative phosphorylation and mitochondrial dysfunction, with lower expression of genes associated with the senescence-protective sirtuin pathway in cluster LCH_1, indicative of a differential metabolic profile in the two clusters (Fig. S4C). There was no difference between LCH_0 and LCH_1 in expression of the LCH signature genes *CSF-1R*, *CD276*, *LMNA* or *PAK1* but upregulation of lipid presentation by CD1 in LCH_0 (Fig. S4C, 4D). Next, we assessed regulatory network activity using SCENIC, which suggested two major regulons that we then mapped back to the UMAP (Fig. S4E). Higher levels of activity of regulons related to the immune system (such as STAT2 and FOXP3) and its developmental processes (IRF4, IRF8) were found in cluster LCH_1, while cluster LCH_0 had higher levels of regulon activity in processes related to both oncogenesis and tumor suppression, that is, ELF1, HDAC2, MYC and p53 regulons (Fig. S4F, G). However, activity in these types of networks was not restricted to cluster LCH_0, as e.g. in cluster LCH_1 higher regulon activity was seen in MTA3 (Fig. S4G), which functions both as an oncogene and as a tumor suppressor (*28*). In summary, this analysis revealed two major LCH subpopulations within lesion LCH cells, which, although closely related, are distinct at the single gene and gene signaling network expression level.

### LCH clusters are related to DC2 and DC3/Monocytes

To probe the relationship of these LCH clusters to known MNP populations we used Connectivity Mapping (CMAP) (*29*) to assess the enrichment of myeloid cell signatures bulk RNA-seq of DC2, DC3 and monocytes (*4*). As the transcriptional signatures of DC3 and monocytes relative to DC2 are highly overlapping and could not be separated, we refer to the DC3-derived signature as DC3/Mono. Strikingly, there was a clear segregation between the two clusters between DC2 versus DC3/mono signatures (Fig. 2B, 2C). In addition, DC2-polarized and DC3/Mono-polarized LCH cell signatures of all their genes, and not only the ones included in CMAP analysis (Fig. 2D, left), selected DC and monocyte clusters, respectively (Fig. 2D, right). As a further test of the mapping of LCH clusters to DC2 and DC3/mono expression profiles, we used the Label Transfer function in Seurat (30) to annotate the LCH cells to a myeloid cell subset, in an unsupervised fashion. Using both bulk signatures (Fig. S4H) and non-LCH myeloid single cell signatures (Fig. S4I) the significant majority of LCH_0 cells we labelled as DC2, and LCH_1 cells as DC3/mono.

We did not find a DC1-like LCH cluster, as previously reported (24), although DC1 were easily found in the non-LCH myeloid cells (Figure 1). DC1-label transferred cells within the LCH population were scattered randomly in the UMAP space (Fig. S5A) and were most easily lost as the Label Transfer function score was increased indicating that their annotation was not robust (Fig. S5B). It is known that Langerhans cells express some transcriptional modules related to DC1, which may account for the sporadic labelling of LCH cells with this signature (31).

### Cross-dataset validation confirms LCH cell polarization towards DC2 and DC3/Monocytes

To extend these observations, we integrated our Smart-seq2 dataset (‘Reference LCH’) with a 10X Genomics dataset of LCH lesions (24), using the Transfer Anchors function of Seurat v3 (30, 32) (Fig. 2E, F). The integrated samples showed an even distribution between patients, and overlapping of LCH cells within each dataset (Fig. 2F). LCH_0 and LCH_1 mapped to discrete regions of the 10X data connected by a mixed region (cluster 2; Fig. 2G). This mixed region showed the most variability between donors and tissue sites (Fig. S4J). Subjecting the 10X data to CMAP analysis revealed a similar significant partitioning between DC2 and DC3/mono signatures, as observed in the SMART-seq2 data (Fig. 2H, 2I).

Integration of the two datasets revealed additional DEG between the two LCH populations, including MHC class II genes in cluster LCH_0 and *FCGBP* and *CD44* in LCH cluster LCH_1 (Fig. 2J). Similar differentially regulated pathways were also highlighted as in our Smart-seq2 data set (Fig. 2K). From the developmental point of view, in line with two different origins, RNA velocity analysis predicted progenitor cells stemming from cluster LCH_0 that were not shared with cluster LCH_1, and the overall trajectory architecture showed a continuum of states between LCH_0 and LCH_1 (Fig. S6) (33–36) (Methods). Next, we were able to assess mutation status of DC2-like (HLA-DQ^+/++^) and DC3/Mono-like (CD14^+^) LCH cells from bulk sorted LCH lesions, and confirmed that they both were heterozygous for *BRAF^V600E^*, while other lesion MNPs were negative for the mutation (Fig. 2L, Fig. S7). HLA-DQ and CD14 were chosen because they are differentially expressed by DC2 and monocytes, respectively (3–5). For the genetic analysis we gated on the extremes of expression, aiming to achieve the highest enrichment of each subset.

### Two LCH subpopulations are found in the tumor microenvironment and receptor-ligand analysis implicates cooperativity between lesional cells

**Fig. 3:**
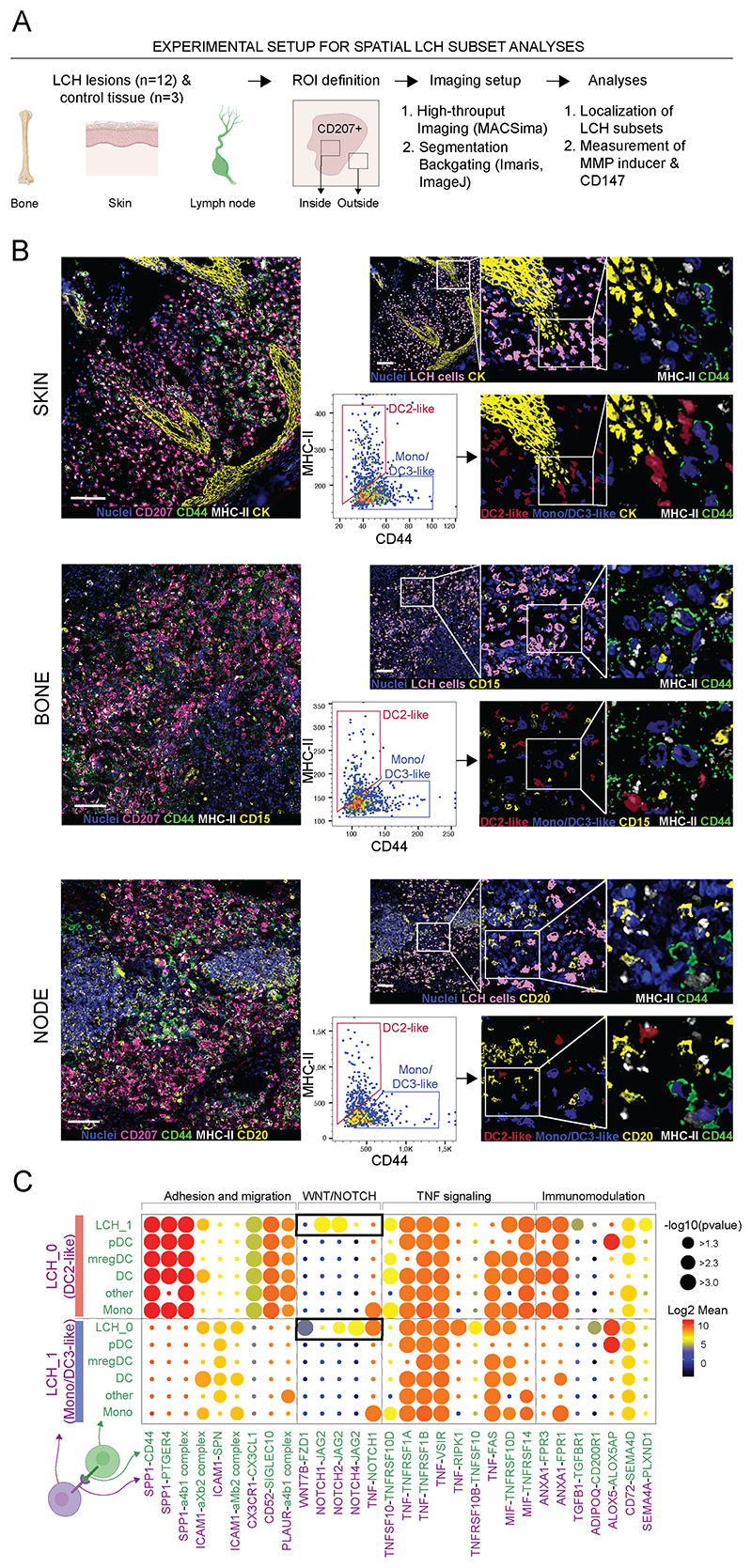
LCH subpopulation spatial distribution and receptor-ligand interactions. (A) Schematic illustration of experimental approach. (B) LCH cells (pink mask, upper panels) from skin, bone and lymph node tumors identified based on CD207 expression using Imaris, and two LCH subpopulations defined in FlowJo gating on MHC-II positive (DC2-like LCH cells, red mask, lower panels) and CD44 positive (Mono/DC3-like LCH cells, blue mask, lower panels) cellular units, backgated onto the original images. MHC-II is presented in white, CD44 in green, CD207 in pink, and nuclei (upper panels) in blue; CK, CD20, CD15 in yellow; scale bar indicates 100 μm. (C) Dot plot of ligand-receptor interactions in LCH_0 (DC2-like) and LCH_1 (Mono/DC3-like) and other lesional cells performed on Smartseq-2 data; P values are indicated by circle size, scale on the right; the means of the average expression level of interacting molecule 1 (green, below) in cell population 1 (violet, left) and interacting molecule 2 (violet, below) in cell population 2 (violet, left) are indicated by color, scale on the right.

Having described transcriptomic heterogeneity of LCH cells, it was important to determine if DC2-like and DC3/Mono-like LCH clusters could be identified in different lesional tissues *in situ*. Using the high-content imaging platform (MACSima; Figure 3A and Fig. S8)) we identified CD207-rich regions of skin, bone and lymph node lesions (Fig. 3B). As expected, CD207^+^ LCH cells clustered in the apical dermal regions of the skin identified by cytokeratin (Fig 3B; CK, yellow, skin). In bone, LCH cells were accompanied by CD66b^+^CD15^+^ eosinophils (CD15, yellow, bone), and in lymph node samples, LCH infiltrated the parafollicular regions (CD20, yellow, node) (Fig. 3B). Using the phenotypic markers MHC class II and CD44, identified from differential gene expression, we observed LCH cells concordant with LCH_0 and LCH_1 clusters (Fig. 3B).

Having identified DC2-like and DC3/Mono-like LCH cells in context of lesional microenvironment, we next sought to determine the potential pathogenic significance of two subsets of LCH beyond their relationship to two different precursors. In order to probe for potential cell-cell interactions, we performed receptor-ligand interaction analysis, focusing on interactions between LCH and non-LCH myeloid cells, and between the two LCH clusters (Fig. 3C). Osteopontin (*SPP1)*, emerged as an important ligand expressed at higher levels on all LCH cells compared to non-LCH cells (Fig. S9). This ligand potentially mediates an interaction between LCH_0 and LCH_1 via CD44 which was specifically enriched on LCH_1 cells. In the other direction, LCH_1 cells were capable of *ICAM1-*based interactions with other myeloid cells, including LCH_0. Predominantly between LCH_0 and LCH_1, we observed Notch interactions, such as the ones mediated by Notch ligand *JAG2* and Notch receptors (*NOTCH 1, 2, 4*) (Fig. 3C). Interactions related to TNF signaling and immunomodulation were predicted for both LCH subpopulations with multiple other cell types (Fig. 3C).

### DC2 promote Langerin induction on DC3 and monocytes through Notch signaling

**Fig. 4:**
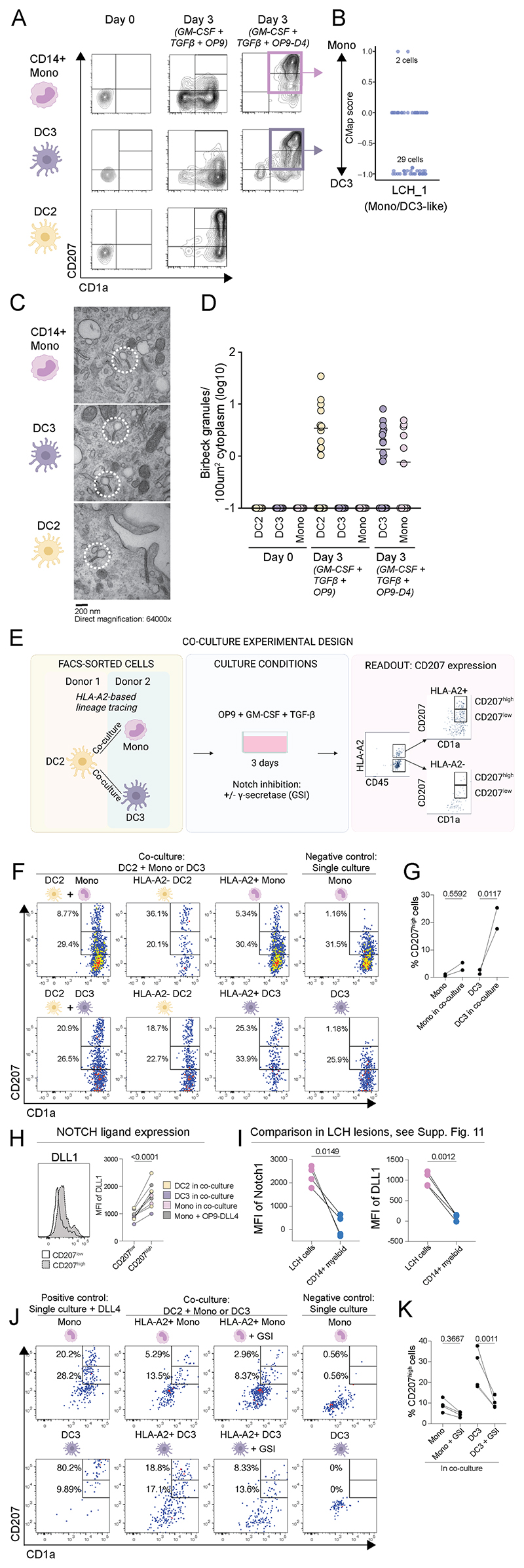
Lineage specific requirements for LCH program and DC2 capacity to promote Langerin induction on DC3 and monocytes through Notch signaling. (A) *In vitro* blood CD14^+^Mono, DC2, DC3; at day 0 and day 3, cultured with GM-CSF/TGFβ +/-OP9-DLL4 (D4). (B) LCH cells from Mono/DC3-like (LCH_1) cluster subjected to CMAP analysis towards Mono and DC3 signatures, obtained from cells cultured under notch ligation conditions (+OP9-D4 from H) (see Fig. S10). (C) Electron microscopy on *in vitro* culture from indicated sources in GM-CSF/TGFβ condition for DC2 and GM-CSF/TGFβ +OP9-DLL4 for DC3 and monocytes; Tennis racket-shaped Birbeck granules encircled. (D) Quantification of Birbeck granules; 0 was replaced with 0.1 for visualization purpose on log scale. (E) Schematic illustration of co-culture experimental design, including HLA-A2-based lineage tracing (first column), culture conditions (second column), and gating strategy after excluding dead cells (third column). (F) Levels of CD1a and CD207 on cocultured FACS-sorted DC2 and monocytes (first column, first row), DC2 and DC3 (first column, second row) presented separately (second and third column), and single culture of monocytes (first row, forth column) and DC3 (second row, forth column). (G) Percentage of CD207^high^ cells among monocytes and DC3 in single culture or in co-culture with DC2 (indicated as “co-culture”). (H) Representative histograms (left) and quantification of NOTCH ligand DLL1 expression on CD207^low^ and CD207^high^ cells in indicated cell populations (DC2,DC3, Mono) from co-cultures (indicated as “in co-culture”) or in single monocyte positive control culture with OP9-DLL4. (I) Notch1 and DLL1 MFI, calculated by subtracting isotype signal (i.e. marker MFI minus isotype MFI) in lesional LCH cells and CD14+ myeloid cells (for gating and details see Fig. S11). (J) Levels of CD1a and CD207 on FACS-sorted monocytes (first row) and DC3 (second row), cultured alone (first and forth column) or in a co-o-cultured with DC2 (second and third column), with NOTCH ligand OP9-DLL4 as positive control (first column), and NOTCH inhibitor γ-secretase (GSI) (third column). (K) Percentage of CD207^high^ cells among monocytes and DC3 in co-culture with DC2 with no inhibition and with NOTCH inhibitor γ-secretase (+ GSI). Statistical differences were assessed using ANOVA with Šídák’s multiple comparisons test in (G, K) and paired t test in (H, I), adjusted P value is specified for (G, K) and P value for (H, I).

It has previously been reported that DC2 readily develop an LCH-like phenotype in response to soluble mediators alone, while monocytes require additional Notch ligation (13, 17, 20, 37, 38). The potential of DC3 to acquire an LCH-program has not been previously documented. We therefore investigated the overall lineage specific requirements for the LCH program *in vitro* under TGF-β1/GM-CSF conditions +/- Notch ligation, in cells from DC2, monocyte and DC3 lineages, sorted from peripheral blood mononuclear cells. This showed that DC3, similar to monocytes and in contrast to DC2, require Notch ligation to gain the LCH phenotype (Fig. 4A). Of note, on a transcriptional level, DC3 culture with Notch ligation, compared to the corresponding culture from monocytes, shared a higher degree of similarity with DC3/Mono-like LCH cells from cluster LCH_1 (Fig. 4B, Fig. S10). In addition, both DC3 and monocyte lineages required Notch ligation for acquisition of Birbeck granules, the presence of which further authenticated the LCH program (Fig. 4C, 4D).

Next, based on: i) Notch dependent receptor-ligand interaction between LCH subsets (Fig. 3C); ii) the presence of both DC2-like and DC3/Mono-like LCH cells within same tissue compartment in LCH lesions revealed by microscopy (Fig. 3B), and iii) differential Notch requirement for DC2 and DC3/Mono lineages in gaining LCH program (Fig. 4A, 4B), we hypothesized that DC2 lineage was able to promote LCH program on DC3 and monocytes, possibly through Notch. To test this hypothesis, we performed HLA-A2-based lineage tracing within co-culture system (GM-CSF/TGFβ/OP9) with sorted DC2 and DC3 and monocytes (Fig 4E). Indeed, both monocytes and DC3, cultured together with DC2, acquired high % of CD207^high^ cells (Fig. 4F), which in the case of DC3 was significant (Fig. 4G). In addition, higher levels of Notch ligand DLL1 were detected on CD207^high^ cells when compared to CD207^low^ cells (Fig. 4H). Additional screening of Notch receptors (Notch 1, 2, 3, 4) and ligands (DLL1, JAG1, JAG2, DLL4) revealed higher expression of Notch 1 and DLL1 on LCH cells (Fig. 4I and Fig. S11) and in vitro-derived LCH-like cells (Fig. S12), Finally, when Notch signaling was inhibited using γ-secretase inhibitor (GSI) lower levels of CD207^high^ cells were detected on both monocytes and DC3 in co-culture with DC2 (Fig. 4J), and this effect was more pronounced in DC3, where the differences were significant (Fig. 4K).

### Higher levels of CD147 are expressed on DC3/Mono-like LCH cells within the tissue microenvironment

**Fig. 5:**
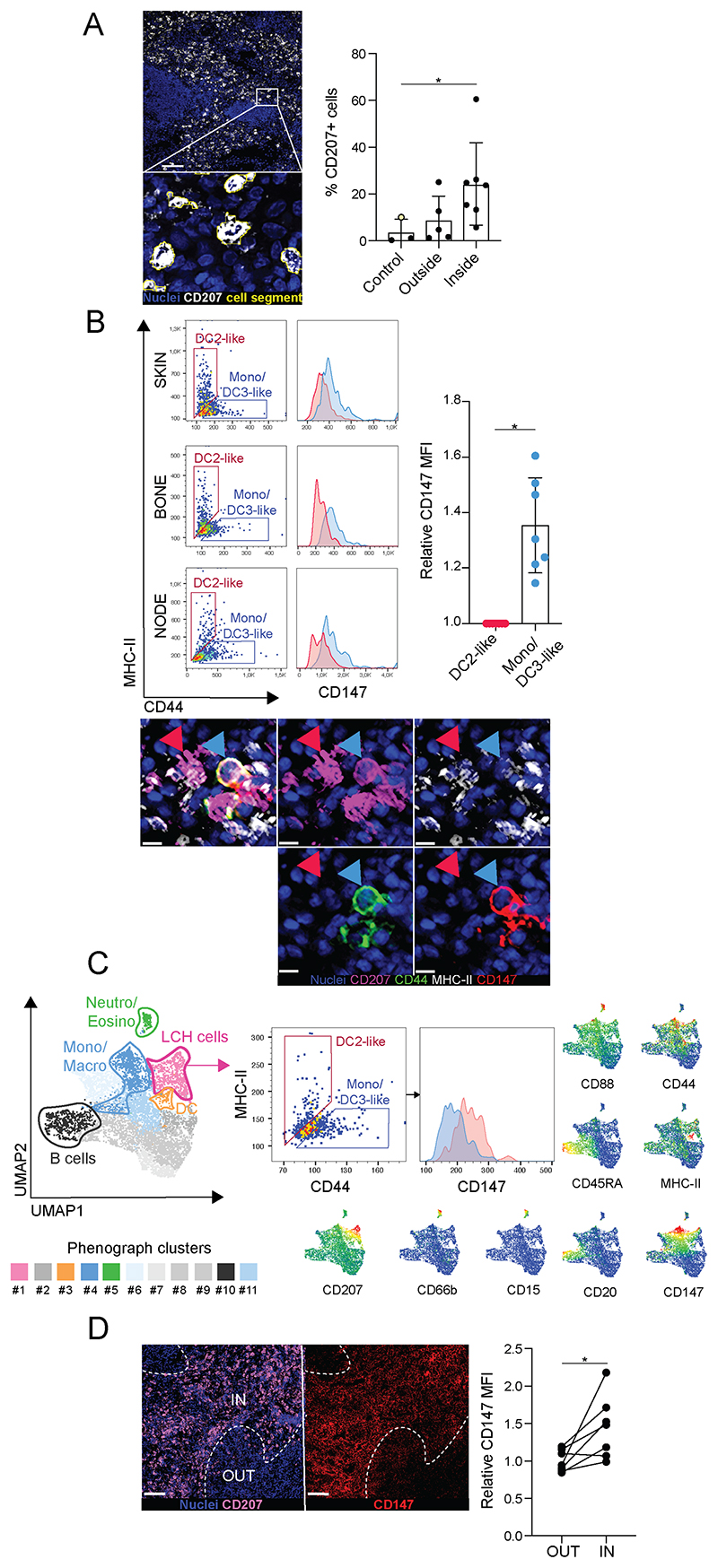
Levels of MMP inducer CD147 in LCH subsets in the lesions. (A) LCH cells identified based on CD207 expression using ImageJ, quantified as percentage of total cells (based on number of nuclei) per image and compared between the images from controls, and LCH outside and inside the tumor. CD207 is presented in white, nuclei in blue, cell segment border in yellow; scale bar indicates 100μm; yellow data point indicates control skin. Statistical evaluation using Kruskal-Wallis test with Dunn’s multiple comparisons. (B) Geometric MFI of CD147 expression and comparison between the subpopulations DC2-like LCH cells (MHC-II positive; red gates, histograms, and arrow heads) and Mono/DC3-like LCH cells (CD44 positive; blue gates, histograms, and arrow heads), defined by FlowJo gating on CD207 positive cellular units from (A). MHC-II is presented in white, CD44 in green, CD207 in pink, nuclei in blue, and CD147 in red; scale bar indicates 10μm. Statistical evaluation using Wilcoxon matched-pairs signed rank test. (C) Unsupervised cell clustering performed using PhenoGraph and UMAP on nuclei segmented cellular units and cell identities established based on median intensity of marker expression presented in plots, from high expression (red) to low (blue). Geometric MFI of CD147 measured in the two subpopulations: DC2-like LCH cells (MHC-II positive, red) and Mono/DC3-like LCH cells (CD44 positive, blue), as defined by FlowJo gating on LCH cells (PhenoGraph cluster #1, pink). (D) MFI of CD147 expression and comparison between CD207 high (IN) and CD207 low (OUT) areas inside lesion, relating values to the mean of CD147 MFI of CD207 low (OUT) of each organ. CD207 is presented in pink, nuclei in blue, and CD147 in red; dashed line shows IN and OUT border; scale bar indicates 100μm. Statistical evaluation using Wilcoxon matched-pairs signed rank test. P values: * p<0.05.

CD147 is an extracellular metalloproteinase (MMP) inducer and a regulator of the tumor microenvironment (39). Significantly higher levels of this antigen were detected in the DC3/Mono-like population (Fig. 5B). Of note, DC3/Mono-like LCH cells (blue arrowheads) were observed near to DC2-like LCH cells (red arrows) in lesions, illustrating that both LCH subpopulations share the tissue compartment they are distributed within (Fig. 5B). To further confirm these findings, unsupervised clustering was performed on cellular units instead segmented by nuclear stain (Fig. 5C). When examining MHC class II expression versus CD44 expression in the LCH PhenoGraph cluster, the two populations were again distinguished and DC3/Mono-like LCH cells again showed higher CD147 levels (Fig. 5C). The expression of these antigens was confirmed using flow cytometry (Fig. S13 and 14). Lastly, CD147 expression was assessed within LCH lesions, comparing areas with low LCH cell frequency (OUT) and areas with high LCH cell frequency (IN), and revealing higher levels of CD147 in the latter (Fig. 5D). Thus, high-content imaging confirmed the differential expression of MMP inducer CD147 on LCH subsets, with higher levels on DC3/Mono-like LCH cells.

## Discussion

In this report, we applied multi-omics and data mining to generate a deep profile of LCH cells and non-neoplastic MNPs from the tumor microenvironment. This revealed two major LCH subsets that phenotypically resemble DC2 and DC3/Monocytes and were detectable *in situ* in diagnostic tissue sections using multi-parameter microscopy, indicating that there may be a dual origin of LCH. Our data support the premise of Notch dependent cross-talk between DC2 and DC3/monocyte lineages, and show that cooperativity between these lineages has the capacity to promote the pathognomonic LCH program. Notably, a similar phenomenon of communication between lineages was recently described in the field of tolerance where IDO-competent DC1 induce regulatory DC2 via metabolic communication (41).

Based on proliferation signatures, higher entropy (34), and several developmental trajectory models (35, 36), we inferred that DC2-like LCH_0 cells do not share the same progenitor as the DC3/Mono-like LCH_1 cluster, consistent with the recently reported distinct origins of DC2 and DC3 (3, 5). It is interesting to note that epidermal LC also segregate into two or more clusters potentially driven by ontogenetic differences, such as a monocyte-like subset and a subset related to antigen presentation, that is a key function of dendritic cells (42). Although LCH cells clearly adopt an LC module of differentiation, and some of the differences between clusters in both LCH cells and LC relate to differential expression of innate or adaptive immune function (e.g. *LYZ* and *HLA-DR*, respectively), there is no a priori reason that these phenomena are related. LCH results from an entirely neoplastic program of differentiation and LCH cells show gross differences in gene expression compared with healthy epidermal LC (16). It is therefore difficult to formally compare the results of heterogeneity described among LCH cells in this manuscript, with the heterogeneity among the normal epidermal LCs recently described by Liu and colleagues (42).

Although the two LCH cell clusters were distinct at the transcriptional and gene network level, antigens defining their phenotypes were distributed on a continuum. This is similar to the markers that define the state of DC2 and DC3 in peripheral blood, which are distinct transcriptional states (4, 23). A parallel phenomenon is seen in DC maturation antigens and expression of the mregDC transcriptional program (25).

Although sorted LCH cells are completely distinct from epidermal LC and other MNPs found in LCH lesions, we found that single-cell transcriptomes required a relatively high sequencing depth, to make this distinction, here achieved using Smart-seq2 protocol (43). Expression of Langerin and CD1a is not completely restricted to LC and LCH cells and care is required to avoid annotation of nonneoplastic lesion MNPs such as cross-presenting DC1 or DCs in the mregDC state, as LCH subclusters (24). The presence of such subpopulations on an individual patient basis cannot be excluded, and further research will be needed to assess inter-individual heterogeneity for related precision medicine approaches. However, from the perspective of LCH heterogeneity across patients, we did not detect additional subclusters among LCH cells either in the Smart-seq2 or in the 10X data sets (from Halbritter *et al*). The accurate identification of *bona fide* LCH-cell clusters is of paramount importance for the development of new immunotherapies and targeted treatments so that any elimination strategy is based on targets specific for the cancer and not the normal MNP at the same site.

While we detected multiple cancer progression/suppression, and likely oncogene-mediated signatures in LCH cells, distinct LC-like imprinting was also evident. *In vitro*, this expression program can be driven by TGF-β1 (44, 45), which the LCH environment is rich in (9, 16, 46–48). However, DC2 and monocyte lineages have different requirements for this transformation. While DC2 readily achieve it under TGF-β1/GM-CSF alone conditions (13), Notch signaling, which is detected in LCH lesions (22), is an additional requirement for Langerin expression by monocytes (13, 20, 37, 38) and was recently implicated in the differentiation of normal epidermal LC (42). Our results demonstrate that DC3 have a similar Notch requirement to monocytes and are potentially closer to LCH_0 than monocyte-derived cells. In terms of localization within the lesions, we observed LCH subsets in close proximity to each other in multiple pathological tissue sites which were examined using high-content imaging. This data together with the evidence for Notch dependent receptorligand interaction between LCH subsets is consistent with the idea that DC2 lineage cells, which readily expression Notch ligands, are able to induce the LCH phenotype on DC3 and monocytes.

A limitation of this study is that we did not define the extent to which LCH cells, and in particular the DC2-like LCH_0 contribute to Notch ligands *in vivo*. Future studies may reveal a role for Notch ligand on stromal and epithelial cells. However, once established, LCH lesions contain few stromal cells and those present appeared to express a lower level of Notch ligands than healthy tissue. In contrast we found a high level of Notch1 and its ligand DLL1 on LCH cells, with transcriptional evidence for Notch signaling within LCH cells (e.g. *HES1*). Interestingly, MMP inducer CD147, which has been implicated as a Notch regulator in one study performed in the context of liver cancer (40), was in our study expressed at higher level on DC3/Mono-like cells. Our data support the premise of Notch-mediated crosstalk between DC2 and DC3/Mono lineages and suggest that a cooperative relationship between these lineages could lead to self-amplifying acquisition of the LCH differentiation program.

Other LCH gene expression characteristics confirmed at the protein level, were higher expression of the protectin CD59 and the immune checkpoint molecule CD276, both potential therapeutic targets in development (49–51). Alongside tumor immune evasion pathways in LCH cells, we also confirmed earlier findings of expression of genes involved in senescence mechanisms (52), which are thought to be at least partially driven by an oncogene (21). Lesion LCH cell persistence through cell cycle arrest and an associated secretory phenotype related to hypercytokinemia is well documented in LCH (53–57). This phenomenon may be explained by activation of a senescence program, which we saw here at the gene (e.g. *FCGBP*, *LMNA*, *CDKN2A*, *PAK1*), protein (e.g. p16, p53, mTOR), pathway (e.g. mTOR, CDC42 signaling), and regulatory network (e.g. ERG1, that is an upstream regulator of p53 tumor suppressor (58)) levels. Nevertheless, elimination of LCH cells, rather than intervention with senescence, appears to be a more straightforward approach, as senescence bypass is an important step in the development of cancer (59), likely protecting LCH neoplasia from more aggressive malignant phenotypes (52, 60, 61).

Importantly, both LCH populations would be potentially susceptible to targeted approaches discussed above.

In conclusion, the data suggest a dual origin model of LCH cells, linked by Notch-mediated cooperativity and provide several new insights into the development of LCH lesions and their potential vulnerabilities to new therapeutic approaches. A single cell dissection of neoplastic histiocytes and their microenvironment allows clear differentiation of functions between lineages, illustrating the potential for a tissue program to be initiated and generated by cross-talk between more than one immune cell type. This phenomenon may emerge in other settings in health and disease as recently illustrated by the example of a tolerance program induced by metabolic signaling between DC1 and DC2 (41).

## Methods

### Pediatric LCH patient recruitment and samples

For cryopreserved lesional cell suspension analysis, pediatric LCH patients were recruited at the Department of Pediatric Oncology, Karolinska University Hospital, Stockholm, Sweden; Division of Pediatric Oncology and Hematology, Skåne University Hospital, Malmö, Sweden; and VIVA-KKH Paediatric Brain and Solid Tumour Programme, KK Women’s and Children’s Hospital, Singapore. Samples were collected during routine diagnostic procedures, either as fine needle aspirations or as surgical specimens. Single-cell suspensions were obtained from the samples by filtering them through 70 μm cell strainer; no digestion was used. Cell suspensions were then preserved in freezing medium containing 10 % DMSO in FCS (Sigma Aldrich, St. Louis, MO, USA). For high-content imaging analysis, diagnostic formalin-fixed, paraffin-embedded (FFPE) LCH biopsies were obtained from Pediatric Hospital Dr. Juan P. Garrahan, Buenos Aires, Argentina. The studies were approved by the Regional Review Board in Stockholm (2009/1937-31/1, 2015/537-32/1, 2019-03956) and the SingHealth Centralised Institutional Review Board (2014/2079). Written informed consents were obtained from patients and their parents.

### Index sorting and pre-processing, quality assessment and analysis of Smart-seq2 single-cell transcriptome data

Cell suspensions from lesions of pediatric LCH patients were indexed-sorted on a FACSAriaIII (BD Biosciences) into 96 well plates containing 3 μl Lysis buffer (Ambion® Thermo Fisher Scientific, Waltham, MA, USA) using a 100 μm nozzle (for details see Supplementary Materials “Index sorting, index data analysis, and Smart-seq2 single-cell data generation”).

Raw reads were aligned to the human reference genome GRCh38 using RSEM program version 1.3.0 with default parameters (62). Gene expression values in transcripts per million (TPM) were calculated using the same RSEM program and the human GENCODE annotation version 25. Quality control, log-normalization, selection of highly variable genes, principal component analysis (PCA) and differentially-expressed gene (DEG) analysis were performed using Seurat R package version 3.1 (63). Across the whole cell population initial quality control was performed to filter cells with high mitochondrial activity (>40%), which left 292 cells. Genes whose expression was not detected in at least 1% (min.cell=3) of all our single-cells were disregarded. The eight most significant PCs were used to generate UMAPs and for unsupervised PhenoGraph clustering (64) (k = 10). Cell type annotation was achieved using the PhenoGraph clustering and flow cytometry data. The Seurat pipeline was then reapplied to the 137 LCH cells using SCTransform normalization (32). The UMAP and SNN clustering (resolution = 0.2) data were generated using the four most significant PCs.

The DEGs were computed using the FindAllMarkers function (test.use = “bimod”) and adjusted pvalue < 0.05 was set as the threshold for DEGs. The DEGs were computed between (1) all PhenoGraph clusters, (2) LCH cells (clusters 2, 3, 5 and 8) and myeloid cells (clusters 1, 4 and 6) and (3) the two SNN clusters of LCH cells. Biological pathways in which DEGs were enriched were defined in comparison (2) using |logFC|> 0.25 and in comparison (3) using |logFC|> 0.1, and were identified using Ingenuity Pathway Analysis (IPA) software.

For details on further analyses see Supplementary Materials “DC2 and DC3/Mono polarization analysis” and “Analysis of 10X single-cell data from Halbritter et al”, for BRAFV600E detection see “BRAFV600E detection using ddPCR”.

### Trajectory inference and single-cell entropy analysis

The TPM table of the 137 LCH cells was subjected to the Monocle 3 analysis workflow (33). The gene expression values were log-transformed and normalized by size using the data pre-processing functions available in Monocle. PCA was then performed followed by projection of the cells onto a two-dimensional space encoding their transcriptional state using Uniform Manifold Approximation and Projection (UMAP) (65) with the first 20 principal components and default parameter settings of n_neighbors = 15 and min_dist = 0.1. The Leiden method (66) with a resolution equal to 0.1 was then used to detect cell clusters. A cell trajectory was drawn on top of the projection using the Monocle reversed graph embedding algorithm.

For pseudotime ordering the cells with the highest entropy rate were selected as a starting point. To find the genes that were differentially expressed along the trajectory, the Monocle graph_test function was used to compute a measure of spatial autocorrelation, the Moran’s I. When this value equals +1 it means that nearby cells on a trajectory have exactly the same expression levels of the gene being tested, while 0 represents no correlation. Having selected the genes with a Moran’s I higher than 0.1, the pheatmap function (R package) was used to visualize these DEGs and the hierarchical clustering algorithm was applied to the genes.

The LandSCENT package v0.99.3 (34) was used to quantify single-cell entropy. First, the scRNA-seq count table were log normalized using the normalize function from the scater package, as recommended by the LandSCENT package. The DoIntegPPI function was then applied to integrate the normalized data with the human protein-protein interaction network (net13Jun12.m) defined by the package. Finally, the CompSRana function was used to compute the signaling entropy rate and estimated differentiation potency. The diffusion map algorithm implemented in LandSCENT package was then used to compare these results to those from Monocle 3.

### Gene regulatory network, cell-cell communication and RNA velocity analysis

Single-cell regulatory network inference and clustering (SCENIC) analysis was carried out for transcriptome-based construction of global GRNs (regulons) using SCENIC R package (v1.1.2), as previously described (*67*). A regulon represents a transcription factor (TF) together with all its putative target genes. Firstly, potential TF targets were inferred based on the single-cell expression data using GENIE3. Next, the GRNs were built based on co-expression modules, regulons of RcisTarget and TF motif enrichment analysis. Then, GRNs were scored using AUCell to create a regulon activity matrix, which was imported into Seurat R package v3.2.2 (*63*) for downstream analysis. Using the Seurat pipeline, PCA and t-distributed stochastic neighborhood embedding (t-SNE) was performed, respectively, in order to visualize the cells based on their regulon activities. Finally, differentially-active regulons among the cell clusters were identified using the Wilcoxon Rank Sum test, followed by Bonferroni correction to obtain adjusted P values.

The CellPhoneDB 2 (68, 69) was used to analyze ligand and receptor expression in cells to predict cell-cell communications. The TPM table of the sequenced cells was analyzed using the CellPhoneDB method, setting the minimum of cells in a cluster expressing a gene to 10%, otherwise with the default parameters.

Annotations of unspliced/spliced reads were obtained for RNA velocity analysis using the velocyto (35). The Stochastic (Default; mode=‘ stochastic’) and Dynamic (mode=‘dynamical’) modelling for the RNA velocity were performed using the scVelo (36). All analyses and results were obtained using default parameters and default data preparation procedures.

### Analysis of single-cell skin data from database DISCO integrated with LCH data

The skin atlas was downloaded from DISCO (70). Mononuclear phagocytes (MNPs), namely pDC, monocytes, macrophages, cDC1, cDC2, mregDC, and LCs were extracted and reclustered at res = 0.5 using dims = 1:15. The LCs clusters were identified using common LC markers such as CD1A and CD207. The annotation of MNP clusters was confirmed by plotting the dotplots for known marker genes (i.e. cDC1 = CADM1, CLEC9A; mregDC = CCR7, LAMP3; cDC2 = CD1c, FCER1A, CLEC10A; LCs = CD1a, CD207; pDC = IL3RA, GZMB; Monocyte = S100A8, S100A9; Macrophage = C1QC, APOE). Thereafter, we plotted the violin plots for these 3 LCH signature genes (CD59, CD276 and CSFR1) in MNP cluster to check their expression. The skin DISCO data was downsampled to 1000 cells per cell type and FastIntegration (71) was used to integrate the skin DISCO atlas data with inhouse data. Later we compared the expression levels of CD59, CD276, CSF1R in LCH (our data) and LCs (DISCO skin atlas data). We also visualized the expression level of Notch receptors (NOTCH1, NOTCH2, NOTCH3, NOTCH4), Ligands (JAG1, JAG2) and other marker genes [RBPJ, MALM1, HES1, OPN, Fringe genes (MFNG, RFNG, LFNG)] in DISCO data and our data.

### Culture systems, preparation of skin and gut samples, and staining

For culture systems, blood mononuclear cells were obtained from healthy volunteers with ethical approval from Newcastle and North Tyneside 1 Research Ethics Committee (08/H0906/72). CD14+ monocytes, DC2 and DC3 were sorted from peripheral blood mononuclear cells using a FACSAria Fusion (Becton Dickinson). For details see Supplementary materials “Culture systems, inhibition of Notch signaling using γ-secretase, and analysis of bulk RNA-seq data” and “Electron microscopy”.

For skin samples, material was obtained from mammoplasty and breast reconstruction surgeries under ethical approval from the Newcastle and North Tyneside Research Ethics Committee 1 (12/NE/0395). For colon control tissue, treatment naïve children were recruited under approval from the Regional Review Board in Stockholm (2010/32-31/4, 2018/323-31/1), and written informed consents were obtained from patients and controls, as well as their parents. Tissue collection, digestion, and staining was performed as previously described (72). 25-color surface staining was performed as previously described (73) and intracellular Osteopontin staining was performed as explained in “Flow cytometry, phosphoflow and cell sorting”. For further details see Supplementary materials “Skin and gut sample preparation”.

### High-content microscopy

Immunofluorescent analysis of tissue sections was performed using the MACSima™ Imaging Platform (Miltenyi Biotec B.V. & Co. KG, Bergisch Gladbach, Germany), which enables fully automated immunofluorescent labeling and imaging of individual biological samples. For details see Methods section of Supplementary materials:

“Tissue preparation for high-content imaging”, “Automated immunofluorescent labeling with the MACSima™ Imaging Platform”, and “Image analysis”.

### Statistical analyses

Significance for Pathway analysis was defined using Ingenuity Pathway Analysis (IPA) software (QIAGEN, Redwood City, CA, USA) (see Fig. 1G, 2K). The DEGs were computed using the using “bimod” test in Seurat and p-value was adjusted based on Bonferroni correction using all features in the dataset, threshold for an adjusted p-value was set to < 0.05 (see Fig. 1D, 1F, 2D, 2J). For further tests, functions, packages, and thresholds regarding single-cell analyses please see the Table S2. Statistical differences in co-culture experiments and ex-vivo measurements of Notch1 and DLL1 were assessed using ANOVA with Šídák’s multiple comparisons test in (4G, K) and paired t test in (4H, I). Differences in percentages of CD207+ cells in tissue samples were evaluated among the three groups (control tissue, tissues outside lesions and tissues inside lesions), using the Kruskal-Wallis test with Dunn’s multiple comparisons (see Fig. 5A). Wilcoxon matched-pairs signed rank test was used to compare the MFI of CD147 expression in the two LCH subpopulations within the same image (see Fig. 5B), as well as in areas inside and outside lesional infiltrate, within the same image (see Fig. 5D).

## Supplementary Material

Supplementary Material

## Data Availability

Preprocessed single-cell RNA-seq data are available via Gene Expression Omnibus (GEO number: GSE173923). Raw sequencing reads are available with controlled access via establishment of a material transfer agreement to safeguard patient privacy. Publicly-available datasets, software and code are specified in the Table S2.
