# Supplementary material for: NOTCH dependent cooperativity between myeloid lineages promotes Langerhans cell histiocytosis pathology
Source: Sci Immunol. Author manuscript; Available in PMC 2023 Jan 28. (PMC7614120; doi:10.1126/sciimmunol.add3330)
Supplement: Supplementary Material [file EMS160046-supplement-supplementary_Material.pdf]

Supplementary Materials
NOTCH dependent cooperativity between myeloid lineages promotes Langerhans cell histiocytosis pathology

Egle Kvedaraite, Paul Milne, Ahad Khalilnezhad, Marion Chevrier, Raman Sethi, Hong Kai Lee, Daniel W. Hagey, Tatiana von Bahr Greenwood, Natalia Mouratidou, Martin Jädersten, Nicole Yee Shin Lee, Lara Minnerup, Tan Yingrou, Charles-Antoine Dutertre, Nathan Benac, You Yi Hwang, Josephine Lum, Amos Hong Pheng Loh, Jessica Jansson, Karen Wei Weng Teng, Shabnam Khalilnezhad, Xu Weili, Anastasia Resteu, Tey Hong Liang, Ng Lai Guan, Anis Larbi, Shanshan Wu Howland, Henrik Arnell, Samir EL Andaloussi, Jorge Braier, Georgios Rassidakis, Laura Galluzzo, Andrzej Dzionek, Jan-Inge Henter, Jinmiao Chen, Matthew Collin, Florent Ginhoux

1. Methods
2. Fig. S1: Delineation of LCH cells and other lesional mononuclear phagocytes (related to Fig. 1).
3. Fig. S2: LCH lesion analyses on regulatory network level (related to Fig. 1).
4. Fig. S3: CD276, CD59, and CD115 expression (related to Fig. 1).
5. Fig. S4: LCH cell heterogeneity and cross-data set validation (related to Fig. 2).
6. Fig. S5: Modelling of DC1 annotation robustness using the Label Transfer function (related to Fig. 2).
7. Fig. S6: Developmental trajectory analyses (related to Fig. 2)
8. Fig. S7: BRAFV600E detection in LCH subsets and other lesional mononuclear myeloid cells (related to Fig. 2).
9. Fig. S8: MACSima analyses (related to Fig. 3)
10. Fig. S9. Validation of Osteopontin (OPN) on protein level (related to Fig. 3).
11. Fig. S10: *In vitro* signature for DC3 and monocytes (related to Fig. 4).
12. Fig. S11. Notch in LCH lesions and skin (related to Fig. 4).
13. Fig. S12. Notch expression in culture (related to Fig. 4).
14. Fig. S13. HLA-DP, CD74, CD44, CD147 expression in LCH subsets (related to Fig. 5).
15. Fig. S14. CD44, HLA-DR, HLA-DQ levels on the whole LCH cell population.
16. Table S1: Patient characteristics.
17. Table S2: Materials, antibodies, reagents.

Methods

Index sorting, index data analysis, and Smart-seq2 single-cell data generation

The indexed (FACS) data were subjected to unsupervised clustering using tSNE and PhenoGraph algorithms (see Fig. 1B), and cells that qualified for scRNA-seq (Smart-seq2) were further subjected to manual gating to verify their cellular identity (see Figures S1A, B), using Seqgeq software (BD Biosciences). Single-cell cDNA libraries were prepared using the Smart-seq2 protocol with the following modifications: (1) 1 mg/mL BSA in Lysis buffer; and (2) 200 pg cDNA with 1/5 reaction of Illumina Nextera XT kit (Illumina, San Diego, CA, USA). The length distribution of the cDNA libraries was monitored using a DNA High Sensitivity Reagent Kit on the Perkin Elmer Labchip (Perkin Elmer, Waltham, MA, USA). All samples were subjected to an

indexed paired-end sequencing run of 2x151 cycles on an Illumina HiSeq 4000 system (Illumina, San Diego, CA, USA), with 293 samples/lane.

DC2 and DC3/Mono polarization analysis

The gene expression signatures of DC2 (CD5+ DC2 DEGs) and DC3/Mono (CD5- CD163- DC3 DEGs / CD163+CD14- DC3 DEGs / CD163+CD14+ DC3 DEGs) were derived from a previous human bulk RNA-seq report (Halbritter et al, Cancer Discov, 2019). The identified 173 and 212 DEGs for each population respectively were submitted to Connectivity Map (CMAP) for gene set enrichment analysis of our expression data of cells from LCH clusters from Smart-seq2 dataset (only genes that were expressed in at least 10% of cells were used for the enrichment). The DEGs were computed between the cells which were mostly DC2 and the cells which were mostly DC3/Mono according to CMAP analysis.
To confirm the findings from CMAP analysis, the Label Transfer algorithm from Seurat v3 was used to classify DC2 and DC3/Mono through the projection of a reference dataset (bulk RNA-seq (1) and single-cell RNA seq (2) from a previous report (Halbritter et al, Cancer Discov, 2019)) onto our Smart-seq2 dataset. Both reference and query datasets were log normalized and 3000 highly variable genes for (1) and 16000 for (2) were selected. After finding anchors, the TransferData function was applied to obtain the prediction. Chi^2 test was used to compare the fraction of DC2 and DC3/Mono cells contained in each cluster to the random situation.

Analysis of 10X single-cell data from Halbritter *et al*

The Seurat package v3.1 was employed to integrate the Smart-seq2 dataset and a previously published 10X data set from Halbritter et al, Cancer Discov, 2019. Cells with >40% mitochondrial gene counts and unique feature counts <200 were filtered out. Then, the remaining cells data from both datasets were log normalized using the SCTransform normalization with the default parameters. Cell anchors in the datasets were found using the Smart-seq2 dataset as a reference and subsequently used for batch integration. To visualize the integrated data, PCA was performed on the scaled integrated gene expression matrix followed by UMAP and SNN clustering on the significant PCs. Then the above pipeline was reapplied to just the three clusters containing the LCH cells from the Smart-seq2 dataset, giving rise to three new LCH-like clusters.
Using the same DC2 and DC3/Mono signatures and the same pipeline described above, a CMAP analysis of these clusters containing LCH cells from Smart-seq2 and 10X datasets was carried out. The DEGs were computed between the cells which were mostly DC2 (LCH-like cluster 0) and the cells which were mostly DC3/Mono (LCH-like cluster 1). Pathways enriched by DEGs using |logFC|> 0.25 were identified using IPA software. The integrated dataset containing the 3 LCH-like clusters was used as input to run the Monocle 3 analysis with the same process and parameters as above.

Tissue preparation for high-content imaging

A series of FFPE LCH biopsies samples was selected. Normal lymph node, bone marrow and skin were used as control samples and were purchased as FFPE tissues from ProteoGenex (ProteoGenex, Inglewood, CA, USA). The samples were cut into 3 µm thick sections and dried overnight at 40°C before dewaxing in xylene

and rehydration in a graded alcohol series. Epitope retrieval was performed in TEC-buffer (pH9) at 98°C for 20 minutes using a heating chamber (Lab Vision™ PT-Modul, Thermo Fisher Scientific, Waltham, MA, USA). The sections were stored in MACSima™ Running Buffer (Miltenyi Biotec B.V. & Co. KG, Bergisch Gladbach, Germany). Nuclear staining was performed with Hoechst (Sigma Aldrich, St. Louis, MO, USA). For identification of regions-of-interest samples were labeled with an anti-CD207 primary antibody (titer 1:200, clone 929F3.01 from Origene, Rockville, MD, USA) which was detected using a phycoerythrin (PE)-conjugated secondary antibody (anti-rat IgG2a, Miltenyi Biotec B.V. & Co. KG, Bergisch Gladbach, Germany) followed by automated imaging using the MACSima™ Imaging Platform (Miltenyi Biotec B.V. & Co. KG, Bergisch Gladbach, Germany).

Automated immunofluorescent labeling with the MACSima™ Imaging Platform
The system operates by iterative fluorescent labeling, image acquisition, and signal erasure, using fluorochrome-conjugated antibodies. Here, LCH tissue samples and control samples were analyzed for the expression of multiple markers. Immunofluorescent labeling was performed using phycoerythrin (PE)-conjugated antibodies (details in the Supplementary Table 2). Two regions per sample were selected for analysis based on the expression of CD207, representing regions inside and outside the LCH lesion within the same tissue, with a high frequency of CD207 expressing cells and with low or no expression of CD207, respectively. Non-specific background labeling was blocked using an FcR-blocking reagent (Miltenyi Biotec B.V. & Co. KG, Bergisch Gladbach, Germany).

Image analysis
LCH cells were segmented based on intensity of membrane expression of CD207 using ImageJ. Grey scale images were first filtered and thresholded before processing with MorphoLibJ, a collection of plugins for ImageJ. The resulting LCH-mask was overlaid onto raw tiff images and median pixel intensity of markers was measured for each cell independently. Percentage of LCH cells was defined as percentage of total cells in the image, i.e. number of LCH cells (based on CD207 expression) per total number of cells (based on Hoechst staining) (see Fig. 5D). For measurement of CD147 expression in the LCH subpopulations, data for all individual LCH cells, segmented as described above, were imported into FlowJo v10.5.3 (BD Biosciences) and relative expression was calculated by comparing geometric mean fluorescence intensity (MFI) of CD147, quantified in the LCH_0 (MHC-II^{high}CD44^{low}) and LCH_1 (MHC-II^{low}CD44^{high}) subpopulations by gating them based on CD44 and MHC-II expression (see Fig. 5E). For measurement of CD147 MFI inside and outside areas within the same image, the abundance of CD207+ cells (i.e. number of LCH cells / total number of cells (Hoechst)) was defined per unit area and mean CD147 grey scale intensity was measured over the whole area after pixel outlier removal (radius 5, threshold 50) using ImageJ (see Fig. 5G).
In addition, a second LCH cell segmentation method was used: cell surfaces were created in Imaris (v8.4.2) using the "Surface" function, based on the intensity of the CD207 channel. Smaller surfaces were then consistently filtered out across different samples to select for LCH cells. Identity of cell surfaces was determined based on gating of LCH cell subsets LCH_0 and LCH_1 in FlowJo FlowJo v10.5.3 (BD Biosciences) and backgated onto the image using the Imaris XT function as previously described (Tan et al., Commun Biol. 2018) (see Fig. 5C). To further verity the findings, unsupervised cell clustering was performed using PhenoGraph and UMAP on cellular

units, created based on Hoechst channel intensity using “Surface” function in Imaris. Then, cell identities were established based on median intensity of key marker expression. The LCH PhenoGraph cluster was subjected to gating of LCH subpopulations in Flowjo as described above, and CD147 MFI was compared between LCH_0 and LCH_1 (see Fig. 5F). Statistical analysis was performed using Prism 8 (GraphPad, San Diego, CA, USA), using tests specified in the Methods methods section “Statistical analyses”.

Culture systems, inhibition of Notch signaling using γ-secretase, and analysis of bulk RNA-seq data

Ten-thousand cells were cultured in RPMI 1640 with 10% heat-inactivated fetal calf serum (Sigma), 2mM L-glutamine (Sigma) and 50 U/ml penicillin (Sigma). Stromal OP9 cells with or without expression of the Notch ligand Delta-like 4 (OP9 or OP9-DLL4) were provided by Juan Carlos Zúñiga-Pflücker (Sunnybrook Research Institute, Department of Immunology, University of Toronto, Ontario, Canada).
For experiments using feeder layers, 5,000 OP9/OP9-DLL4 cells were plated 24 hours before the sort. Supplements were added at the following concentrations: 50 ng/mL GM-CSF, 10 ng/mL TGFb +/- 5uM gamma secretase inhibitor (Merck). For co-culture experiments, donors with opposing HLA-A2 status were used in order to determine the originating donor by flow cytometry after 3 days of culture at 37°C. Flow cytometry analysis was performed on a FortessaX20 Cytometer (Becton Dickinson) using appropriate isotype controls. Antibodies were CD1a AF700 (HI149); CD45 APCCy7 (2D1), HLA-A2 BV510 (BB7.2) (all from Biolegend) and Langerin PE (DCGM4) (Beckman Coulter).
For bulk RNA-seq data, raw reads were aligned to human reference genome GRCh38 using STAR-2.7.5a with default parameter settings. Read count per gene was generated using featureCounts functions of subread-2.0.1-Linux-x86_64 package and GRCh38 gencode.v34.annotation.gtf downloaded from https://www.gencodegenes.org/. Count per million reads (CPM) table was generated using cpm function of R package ‘edgeR’. R package ‘DESeq2’ (v1.24.0) was used to obtain differentially expressed genes with adjusted p. value < 0.05 between different groups. Top DEGs with the highest log2 fold change were then plotted using their scaled values on heatmaps using R package ‘pheatmap’ (v1.0.12).

Skin and gut sample preparation

Skin samples were cut into 2 cm wide strips and excess fat was removed with scalpels. Epidermis and upper dermis layers were trimmed off using Goulian skin graft knife with WECPREP BLADES and a .008 blade guard (Teleflex Surgical). Cut sections were floated in a petri dish, epidermis upwards, in RPMI + Dispase II (Gibco; Stock 100U/ML, used at 1:100) and incubated at 37°C for 1 hour. Epidermis was peeled from dermis and both were digested in RPMI +10% FCS + Collagenase for ten hours. (Worthingtons Type IV; Stock 160mg/ml; 1:150 for dermis and 1:200 for epidermis). Cells were strained through 100um strainers, and washed in RPMI+10%FCS. Cells were incubated with mouse IgG (Sigma; used at 50ng/ml) for 10 minutes to inhibit non-specific staining and stained with flow cytometry antibodies for 30 minutes.
For gut samples, pinch biopsies were obtained from treatment naïve children undergoing their first diagnostic colonoscopy for suspected IBD, and were matched with a blood sample for PBMC analysis, collected at the same day, prior to colonoscopy. Biopsies were digested using 250 μg/ml DNase and collagenase II

(Sigma) at 37 °C with magnetic stirring at 650 rpm for 25 min, followed by filtering through a 70 µm cell strainer.

Electron microscopy
Cells were fixed according to standard protocols in 2% glutaraldehyde, and then pelleted, dehydrated, and fixed in resin (all from TAAB Laboratory, Aldermaston, United Kingdom). Ultrathin sections were cut with a diamond knife on an RMC MT-XL ultramicrotome (RMC Boeckeler, Tucson, AZ) and examined with a Philips CM100- Compustage (FEI) Transmission Electron Microscope (Philips, Amsterdam, Netherlands). Images were collected with an AMT-CCD camera (Deben, Bury St Edmunds, UK).

BRAFV600E detection using ddPCR
The QuantStudio 3D Digital PCR 20k Chip system was used to run the Thermo Fisher Taqman Hs000000004_rm (BRAFV600E) assay according to the manufacturer's instructions. Each sample was run on two separate chips and each chip was analyzed in two orientations on a QuantStudio 3D Digital PCR System, giving a total of four readings per sample. Data were analyzed using the QuantStudio 3D AnalysisSuite. Thresholds were set using universal standards for all fractions from each sample to call only single positive wells for FAM and VIC. The ratio of mutant to wild type BRAF copies/µL was calculated within each individual chip reading.

Flow cytometry, phosphoflow and cell sorting
Cell suspensions were washed and incubated in 5% heat-inactivated FCS (Sigma Aldrich, St. Louis, MO, USA) for 15 min at 4°C. For extracellular labeling, cells were resuspended in PBS containing 2% FCS and 2 mM EDTA and a mixture of antibodies, and incubated for 30 min at 4°C. For sorting, cells were washed twice, kept on ice then until the sort, and resuspended in DAPI (Thermo Fisher Scientific, Waltham, MA, USA) immediately before sorting. To label cells for phosphoflow, extracellular antibody mix was supplemented with Live/Dead Fixable Blue stain (Thermo Fisher Scientific, Waltham, MA, USA), and applied as described above, followed by fixation in 100 µl BD Cytofix Fixation Buffer (BD Biosciences, Cat. 554655) for 15 min at 4°C. Next, cells were permeabilized in 200 µl ice-cold BD Phosflow Perm Buffer III (BD Biosciences, Cat. 558050) for 30 min at 4°C, and subsequent intracellular labeling was performed for 30 min at room temperature. Flow cytometry was performed on a FACSymphony A5 (BD Biosciences), FACSFortessa (BD Biosciences), or FACSAria Fusion Cell Sorter (BD Biosciences), and data were analyzed using FACSDiva 6.0 (BD Biosciences) or FlowJo v10.5.3 (BD Biosciences). Details of antibodies used are provided in the Supplementary Table 2.

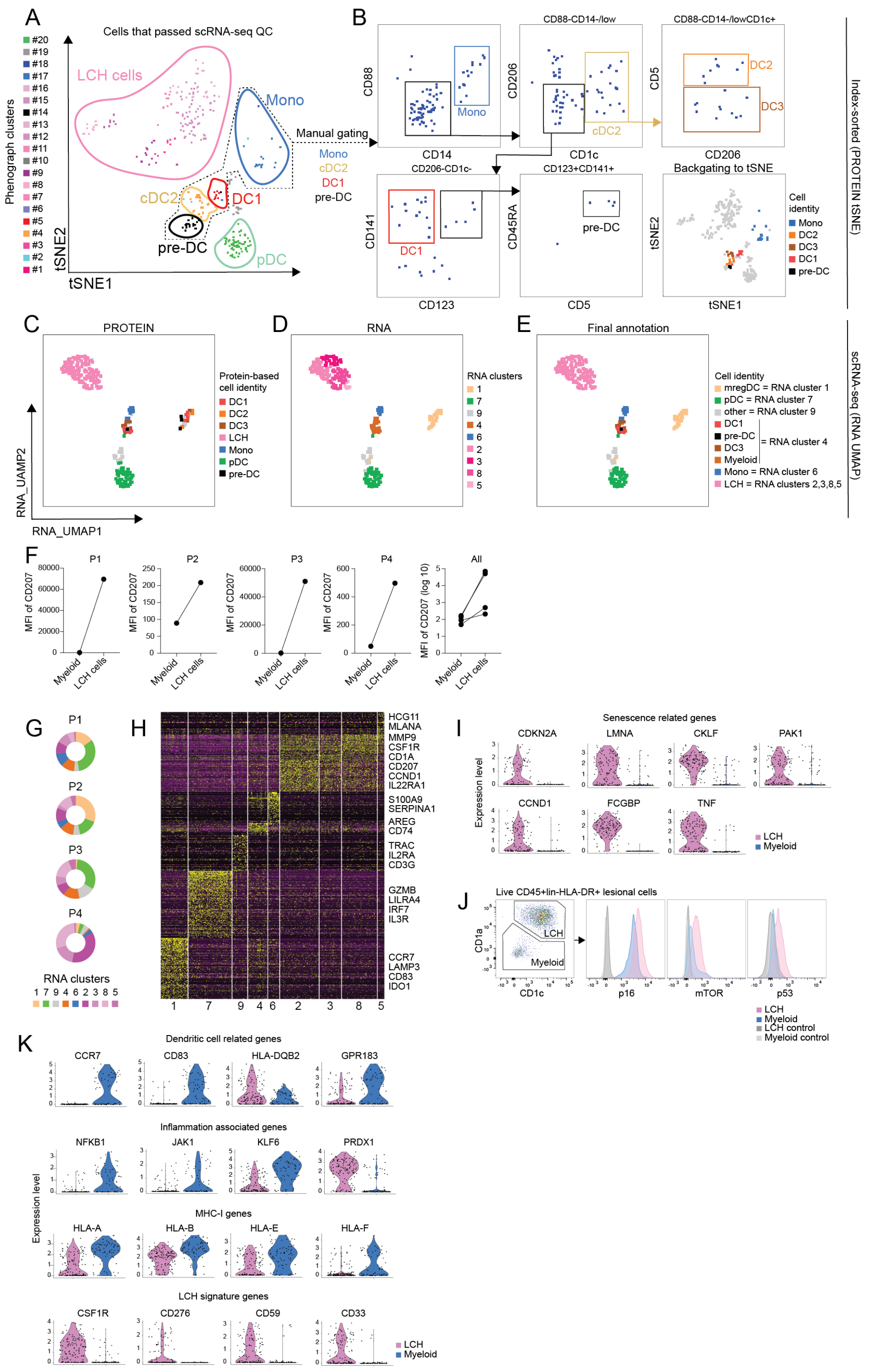

Fig. S1. Delineation of LCH cells and other lesional mononuclear phagocytes. (A) Protein expression analysis of index-sort data that passed scRNA-seq quality control (QC) presented in a tSNE plot with color-coded PhenoGraph clusters; cell identity on protein level established based on expression of signature markers presented in plots in Fig. 1B. (B) Manual gating of protein index-sort data on the selected cells. PhenoGraph clusters from (A), corresponding to pre-DC, DC1, cDC2, and Mono; depicting CD5^{+}DC2, CD5^{-}DC3, CD141^{+} DC1, CD141^{+}CD123^{+} CD45RA^{+}CD5^{+} pre-DC, backgated to the original protein expression based tSNE

plot. (C) scRNA-seq data presented in a UMAP plot, annotated according to the protein expression based cell identities (from A and B). (D) sc-RNA data presented in a UMAP plot with color-coded PhenoGraph clusters (RNA clusters). (E) scRNA-seq data presented in a UMAP plot, annotated based on PhenoGraph cluster-derived cell identities and further specified based on protein data, as follows: LCH cells (clusters 2, 3, 5, 8), mregDC (cluster 1), pDC (cluster 7), other (cluster 9), DC1 (cluster 4), pre-DC (cluster 4), DC3 (cluster 4), Myeloid (the remaining cells from the DC cluster 4), Mono (cluster 6) (also see Fig. 1). (F) CD207 MFI in mononuclear myeloid cells and LCH cells in patients P1-P4 presented separately and together (far right). (G) Relative abundance of RNA expression data-based-cell-clusters in lesional samples from each patient (P1-P4). (H) Heatmap showing relative expression level of significant DEGs between the RNA-based-cell-clusters, with a cut-off of 0.5 log fold change; high to low expression indicated as yellow to dark purple. (I) Violin plots showing relative expression levels of selected DEGs related to senescence in LCH cells (pink) and other mononuclear myeloid cells (blue). (J) Gating strategy used for phosphoflow cytometry of lesional cells; histograms showing mean fluorescence intensity of expression of the indicated proteins in LCH cells (pink), other mononuclear myeloid cells (blue), and labeling with isotype-matched control antibodies (grey and light grey, respectively). (K) Violin plots showing relative expression level of selected DEGs in LCH cells (pink) and other mononuclear myeloid cells (blue).

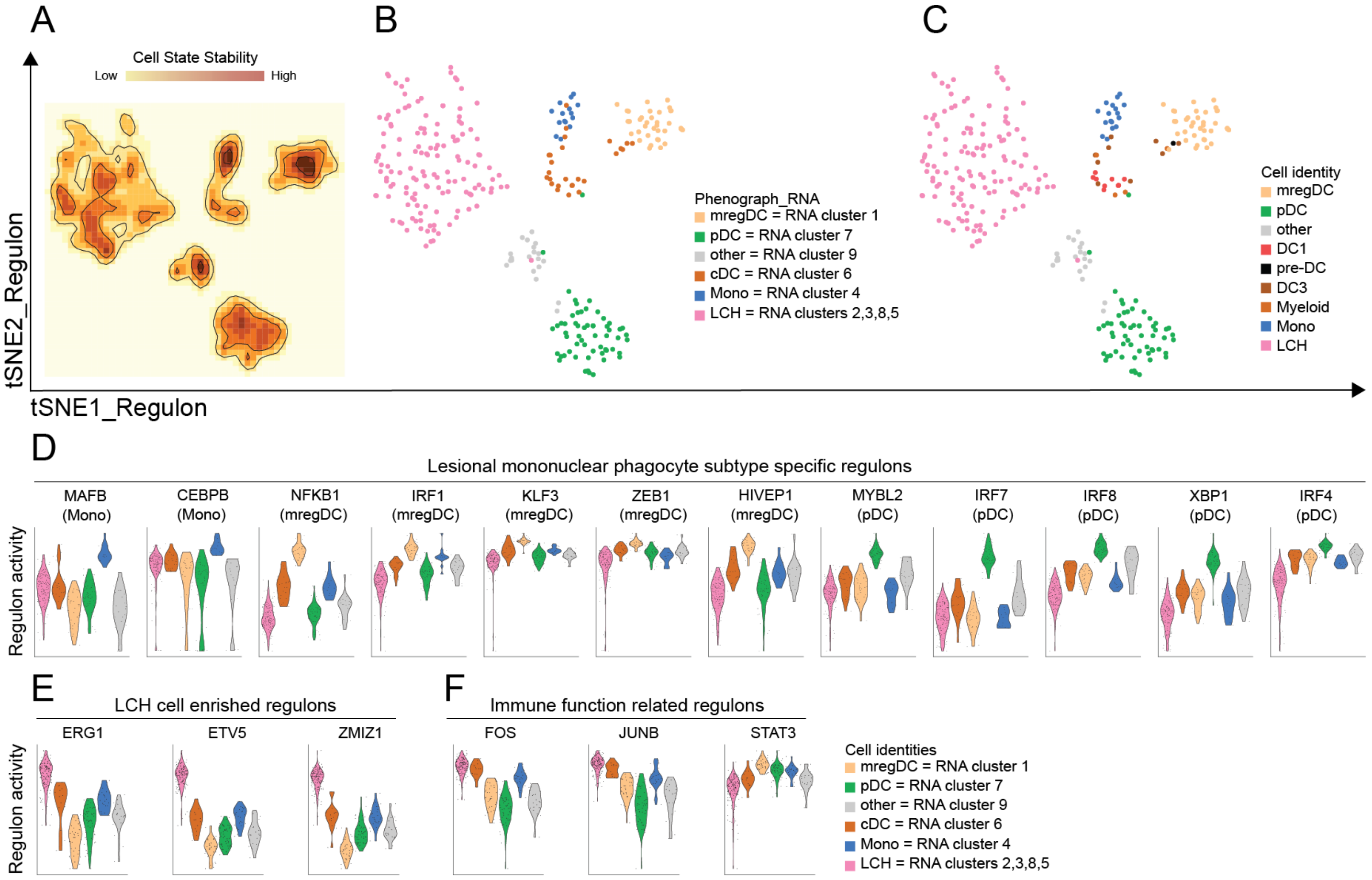

Fig. S2. LCH lesion analyses on the regulatory network level. (A) Cell state stability depicted in a regulatory network (regulon) activity-based tSNE plot. (B) Regulon tSNE with color-coded PhenoGraph clusters (RNA data-based-clusters). (C) Regulon tSNE, annotated based on finalized lesional cell identities (see also Supplement Fig. 1E). (D) Violin plots showing relative activity level of differentially active regulons among the lesional mononuclear phagocytes; the cell subset in which the regulon most active is depicted in the parentheses below the name of the regulon. (E) Violin plots showing relative activity level of selected regulons more active in LCH cells. (F) Violin plots showing the relative activity level of selected regulons involved in immune system signaling.

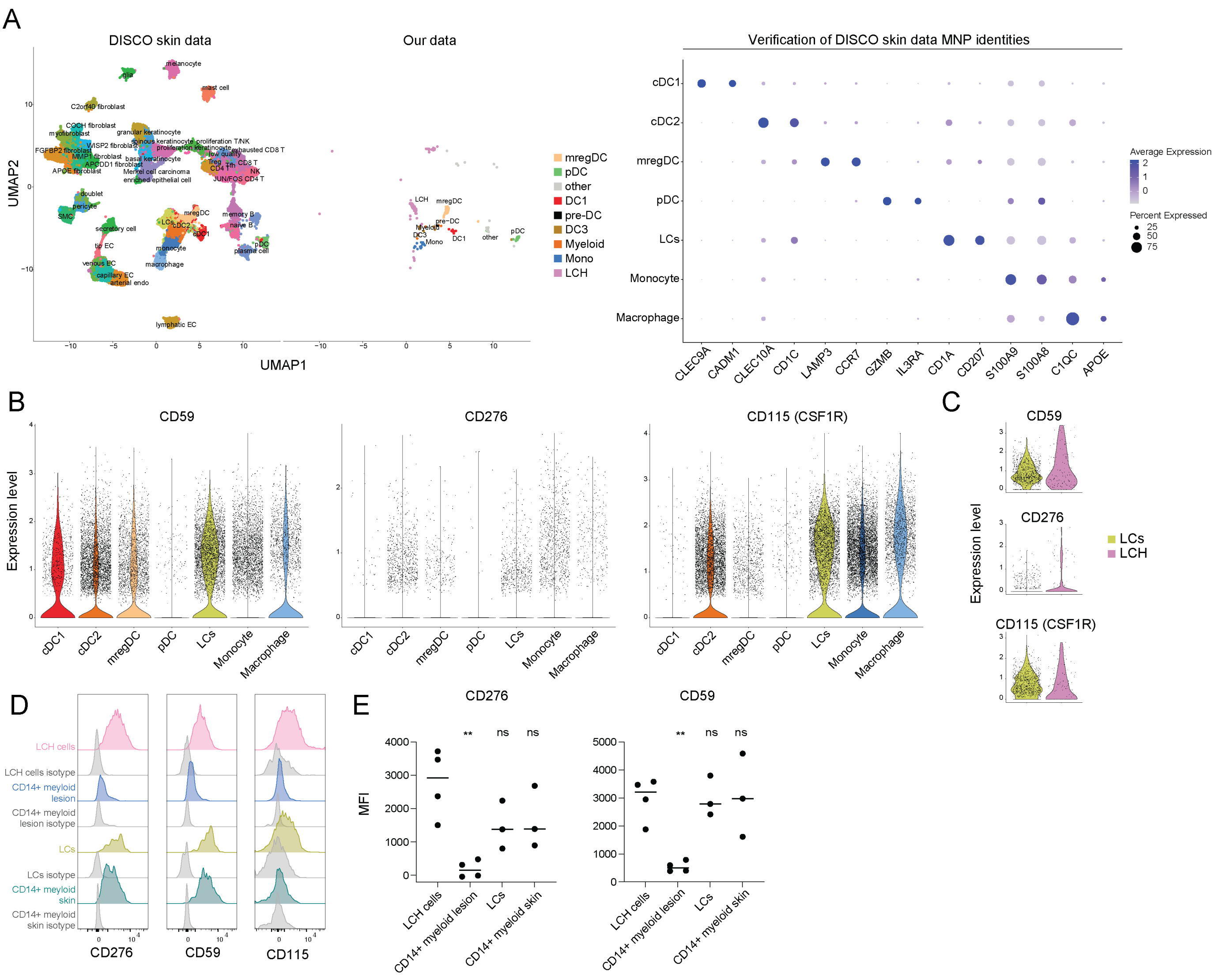

Fig. S3. CD276, CD59, and CD115 expression. (A) DISCO skin samples (left UMAP) integrated with our data (right UMAP); key markers of subsets in the DISCO data presented as a dotplot (left). (B) Levels of markers in the skin, scRNA-seq data, DISCO. (C) Levels of markers in integrated LCs (DISCO) and LCH cells (our data), scRNA-seq data. (D) Levels of markers and isotypes in LCH cells and matched myeloid cells as well as LCs and matched myeloid cells (for gating see Response Figure 1). (E) MFI levels, calculated by subtracting isotype signal (i.e. marker MFI minus isotype MFI). ANOVA with Holm-Šídák's multiple comparisons test for multiple comparisons between LCH lesion data and other conditions, p value : * <0.05, ** <0.01, *** <0.001

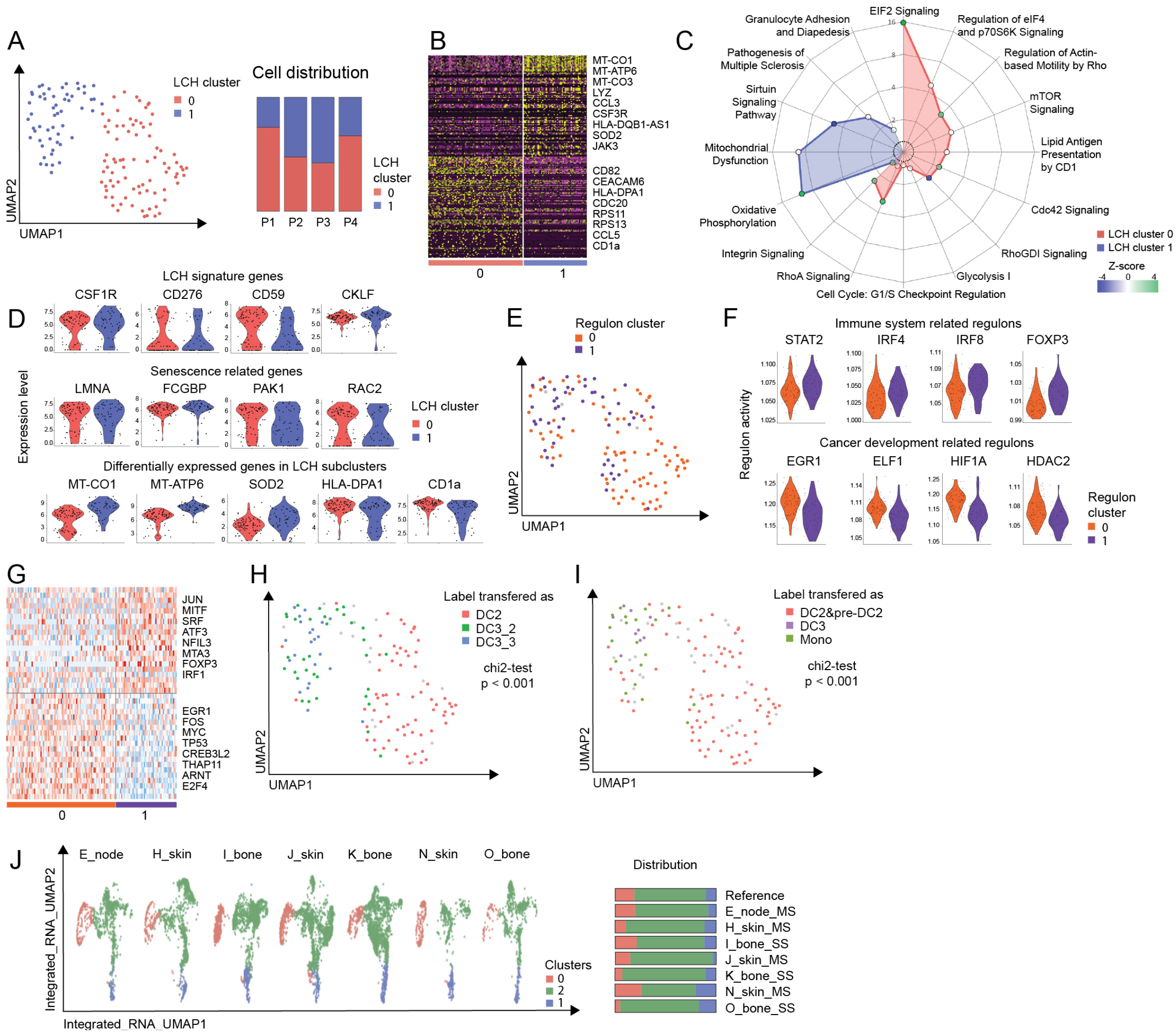

Fig. S4. LCH cell heterogeneity and cross-data set validation. (A) LCH cells subjected to PhenoGraph clustering (indicated as LCH cluster) based on gene expression data, presented in a UMAP (from Fig. 2A) and relative abundance of the LCH clusters in lesional samples from each patient (P1-P4). (B) Heatmap of top 200 DEGs between the two LCH clusters; high to low expression indicated as yellow to dark purple. (C) Ingenuity pathway analysis of the DEGs in the two LCH clusters (red and blue), displayed as a spider web plot showing log (p value) and Z score for each pathway, calculated using DEGs expressed at higher or lower levels in each LCH subset; dashed circle indicates significance level at P<0.05. (D) Violin plots showing relative expression level of LCH-specific genes (upper and middle panels) as well as DEGs between the two LCH clusters (lower panel); presented in LCH cluster 0 and 1 (red and blue, respectively). (E) Regulon PhenoGraph clusters (indicated as Regulon cluster) plotted in a UMAP from (A). (F) Violin plots showing relative expression level of differentially-active regulons in Regulon clusters 0 and 1 (orange and violet, respectively). (G) Heatmap showing relative activity level of top 20 differentially-active regulons between the two Regulon clusters; high to low activity indicated as red to blue. (H) UMAP showing bulk RNA data from Dutertre et al label transfer for DC2, DC3_2 (CD163+CD14- DC3) and DC3_3 (CD163+CD14+ DC3), applied to LCH cells. Distribution of DC2 label-transferred and DC3/Mono-label transferred LCH cells in the two LCH clusters assessed using chi2 test. (I) UMAP showing

single-cell RNA data from *Dutertre et al* label transfer for DC2&pre-DC2, DC3-enriched and monocyte clusters, applied to LCH cells. Distribution of DC2&pre-DC2 label-transferred and DC3-enriched/monocyte-label transferred LCH cells in the two LCH clusters assessed using chi2 test. (J) 10X LCH clusters 0, 2 and 1 (from Fig. 2G) shown in each 10X sample (right) with relative abundance (right).

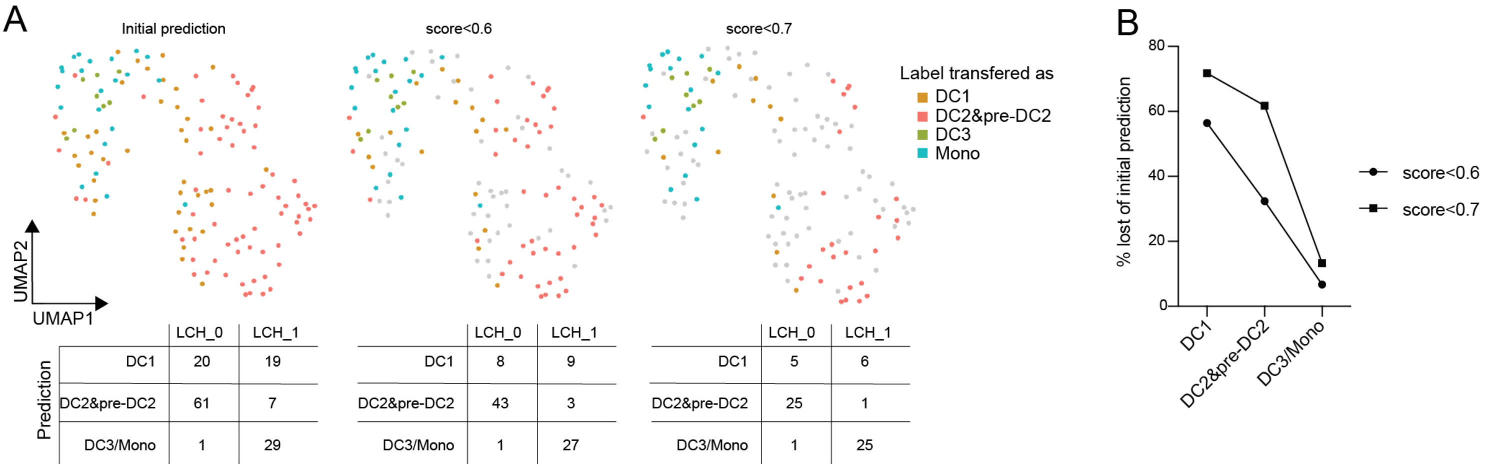

Prediction	Initial prediction LCH_0	Initial prediction LCH_1	score<0.6 LCH_0	score<0.6 LCH_1	score<0.7 LCH_0	score<0.7 LCH_1
DC1	20	19	8	9	5	6
DC2&pre-DC2	61	7	43	3	25	1
DC3/Mono	1	29	1	27	1	25

Fig. S5. Modelling DC1 annotation robustness using the Label Transfer function. (A) Using single-cell data from *Dutertre et al* label transfer for DC1, DC2&pre-DC2, DC3, and Mono clusters was applied to LCH cells, and modeled using indicated scores. (B) Percentage loss of predicted labels using different scores shown for cells identified as DC1, DC2&pre-DC2, and DC3/Mono.

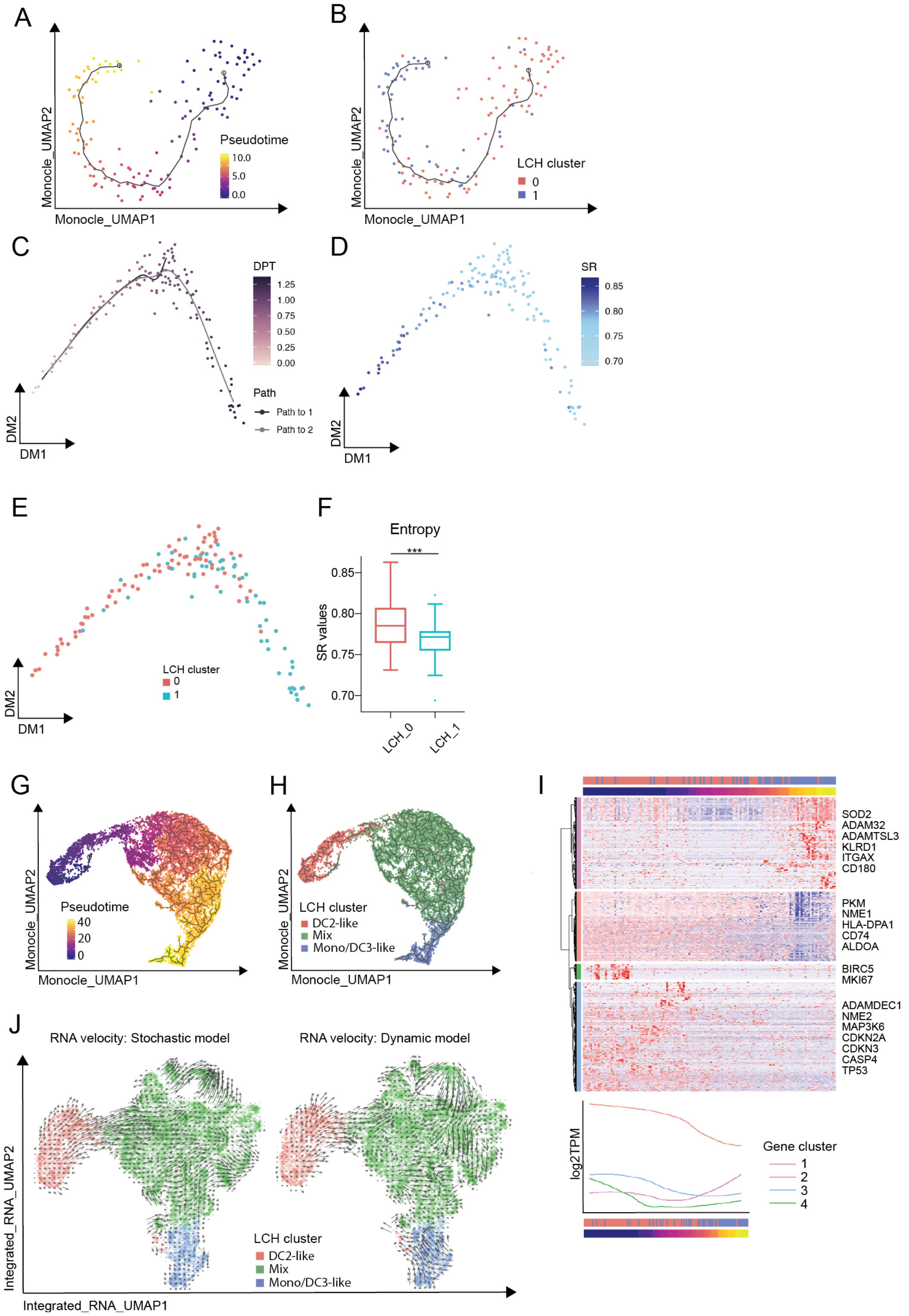

Fig. S6. Developmental trajectory analyses. (A) Smart-seq2 LCH cells in a Monocle UMAP, annotated by pseudotime. (B) Smart-seq2 LCH cells in a Monocle UMAP, annotated by LCH clusters. (C) Diffusion map annotated by diffusion pseudotime (DPT); two paths are indicated in black and grey color, Smart-seq2 data. (D) Diffusion map annotated by entropy rate (abbreviated as SR), Smart-seq2 data (confirms starting point for Monocle in Supplementary Fig. 5A). (E) Diffusion map annotated by LCH cluster identity, Smart-seq2 data. (F) Comparison of entropy rate

between the two LCH clusters, Smart-seq2 data. (G) 10X data defined LCH cells in a Monocle UMAP, annotated by pseudotime. (H) 10X data defined LCH cells in a Monocle UMAP, annotated by 10X LCH clusters. (I) Heatmap showing relative expression level of gene clusters along the trajectory; Smart-seq2 LCH cells ordered by pseudotime, LCH clusters indicated (0 as red and 1 as blue) (upper panel) and variation of expression of gene clusters along the trajectory; LCH cells ordered by pseudotime, LCH clusters indicated (0 as red and 1 as blue), Smartseq-2 data (lower panel). (J) RNA velocity analysis of 10X LCH cells using stochastic and dynamic modelling, annotated by 10X LCH clusters.

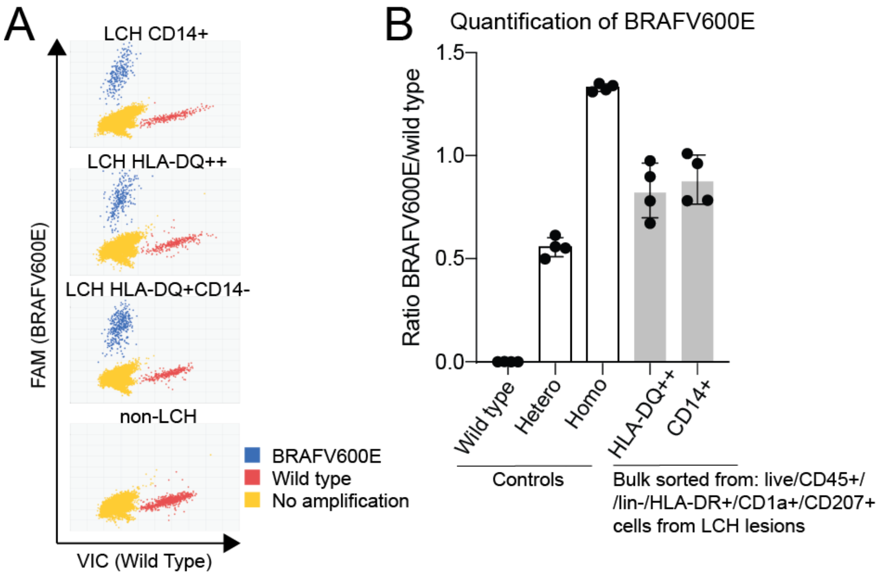

Fig. S7. BRAFV600E detection in LCH subsets and other lesional mononuclear myeloid cells. (A) Raw amplification plots for fluorescence dyes FAM and VIC used for ddPCR, for detection of BRAFV600E and wild type, respectively, in the indicated cell populations. (B) BRAFV600E quantification presented as the ratio of BRAFV600E to wild type in LCH cells as well as wild type, heterozygous and homozygous BRAFV600E cell lines, used as controls.

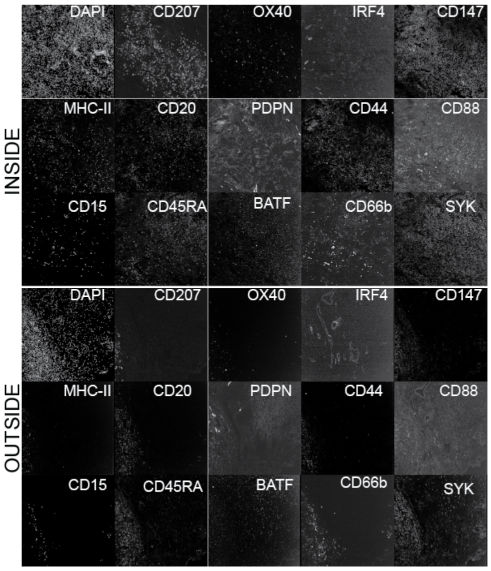

Fig. S8. MACSima analyses. Sequential labeling of tissue sections allows the generation of high-dimensional fluorescence microscopy images inside and outside the tumor.

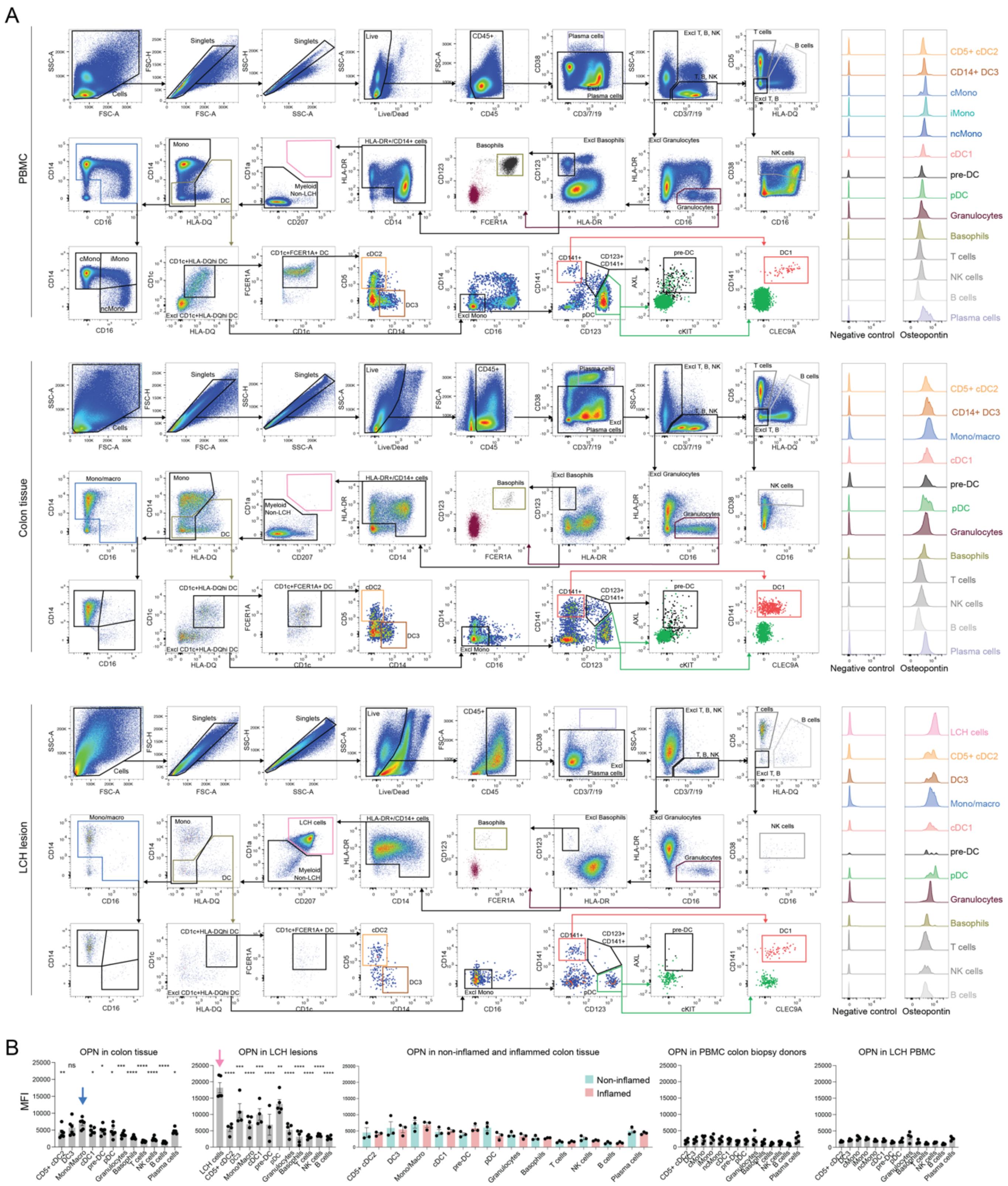

Fig. S9. Validation of Osteopontin (OPN) on protein level. (A) Gating strategy and representative histograms. (B) Quantification of OPN in colon tissue, LCH lesions, between inflamed and non-inflamed colon tissue, as well as PBMCs. ANOVA with Holm-Šídák's multiple comparisons test for comparisons between the subset expressing the highest levels of OPN (Mono/macro in colon, and LCH cells in

LCH lesions) and the remaining subsets, p value: * <0.05, ** <0.01, *** <0.001, **** <0.0001

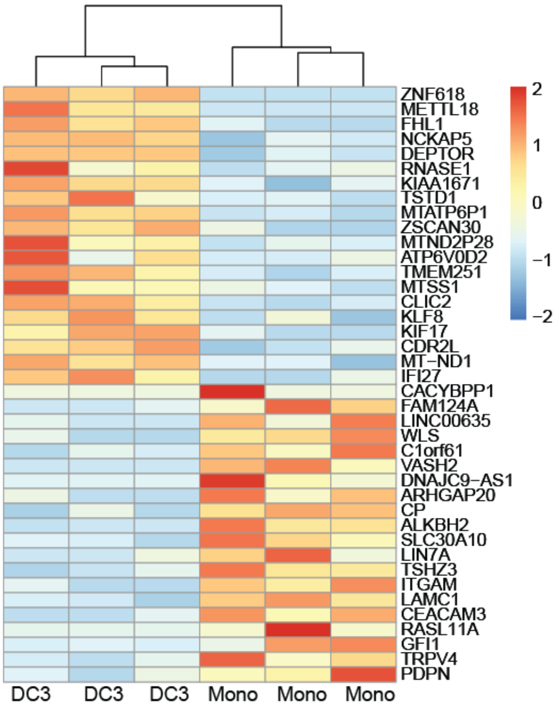

Fig. S10. ***In vitro* gene signatures.** Heatmap showing relative expression level of DEGs between DC3 and Mono from *in vitro* cultures, in the presence of GM-CSF, TGFβ and notch ligation (OP9-D4); used in the CMAP analysis shown in Fig. 4B.

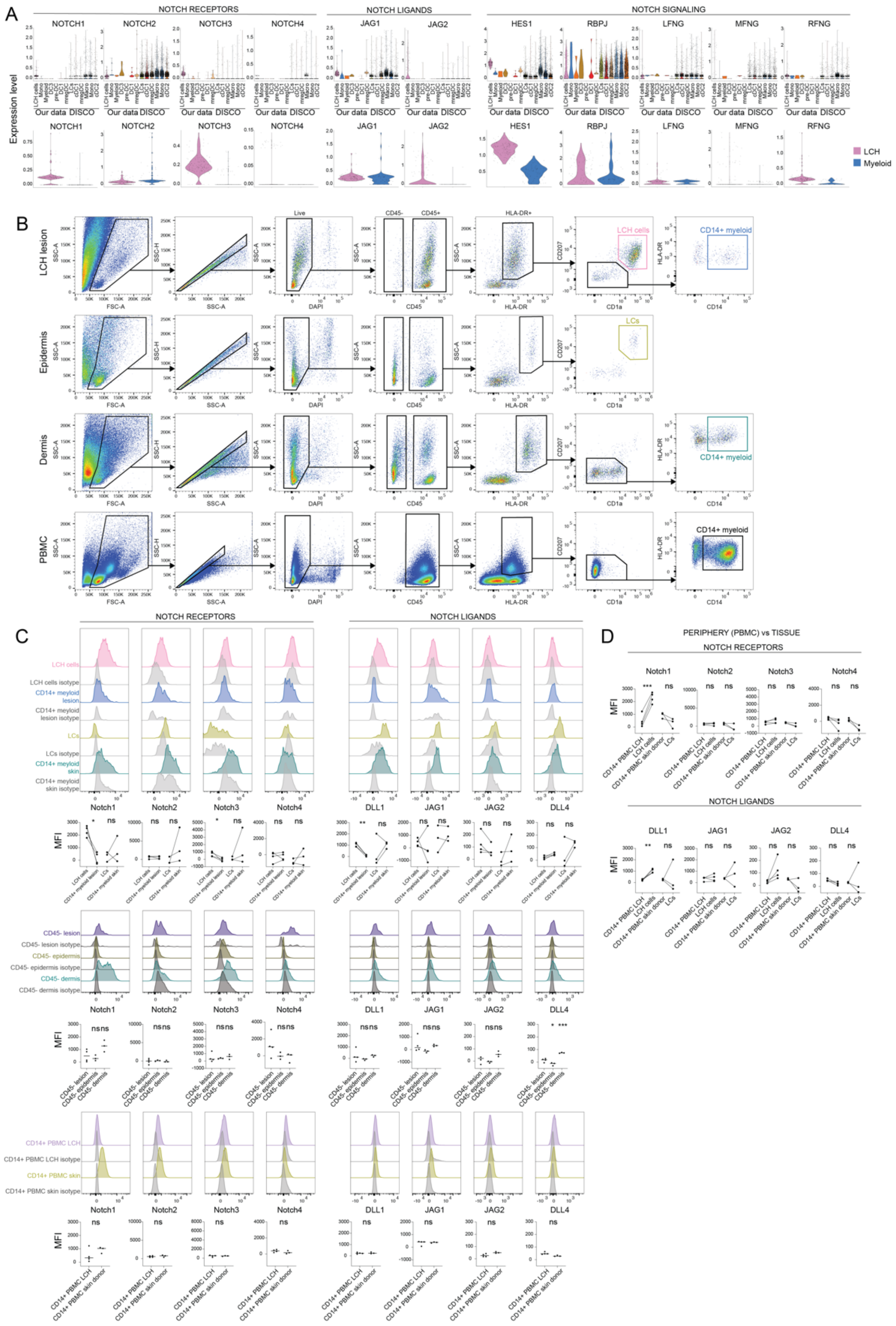

Fig. S11. Notch in LCH lesions and skin. (A) Notch receptors and ligands in single

cell data of LCH lesions integrated with skin samples using data base DISCO (for integration details also see Response Figure 4). (B) Flow cytometry gating strategy. (C) Notch receptors and ligands expression and MFI, calculated by subtracting isotype signal (i.e. marker MFI minus isotype MFI) in tissue (upper and middle panels), and in PBMCs (lower panel). (D) Matched comparison between tissue and PBMCs. Paired T test was used for pair-wise comparisons, and ANOVA with Holm-Šídák's multiple comparisons test for multiple comparisons between LCH lesion data and other conditions, p value: * <0.05, ** <0.01, *** <0.001

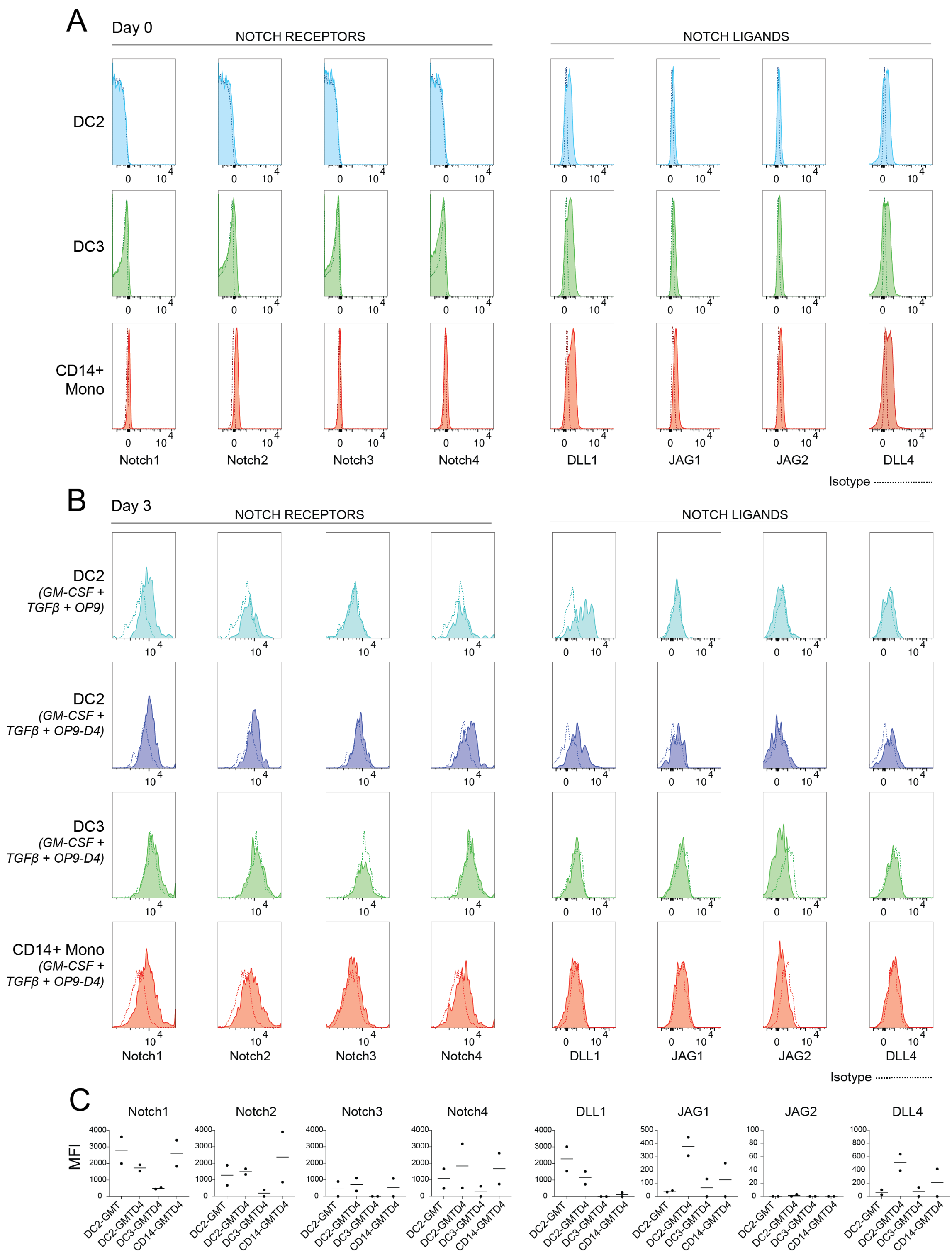

Fig. S12. Notch expression in culture. (A) Notch receptors and ligands in sorted DC2, DC3 and CD14+ Mono prior to culture (Day 0), isotype as dotted line. (B) Notch receptors and ligands in sorted DC2, DC3 and CD14+ Mono post culture (Day 3), isotype as dotted line.(C) Notch receptors and ligands expression and MFI, calculated by subtracting isotype signal (i.e. marker MFI minus isotype MFI), in culture condition with GM-CSF, TGF-b, and OP-9 (GMT) or GM-CSF, TGF-b, and OP-9-DLL4 (GMTD4).

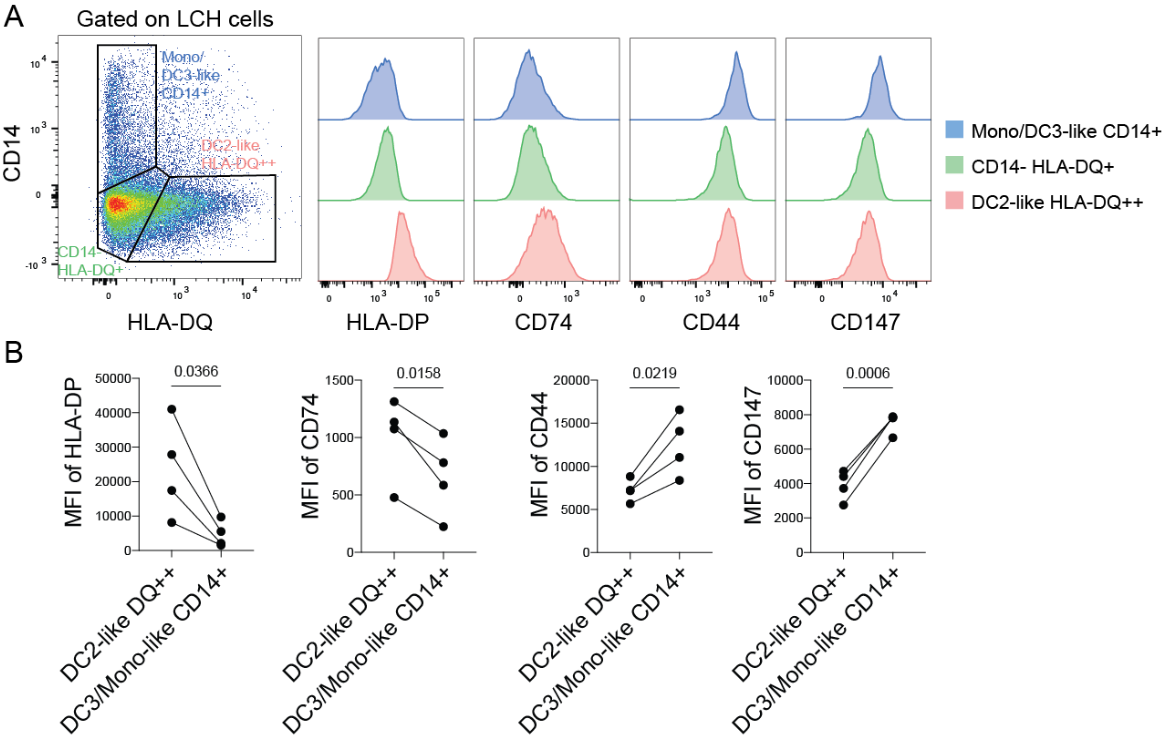

Fig. S13. HLA-DP, CD74, CD44, CD147 expression in LCH subsets. (A) Gating strategy and representative histograms of markers. (B) Quantification of markers among the subsets, gated on the respective ends of the continuum, as shown in A. Of note, while higher levels of CD44 were detected in DC3/Mono-like LCH cells and HLA-DP and HLA-DQ on DC2-like cells LCH cells, all LCH cells express both CD44 and MHC class II (HLA-DR, -DP, DQ). Paired T test was used for pair-wise comparisons, p value indicated.

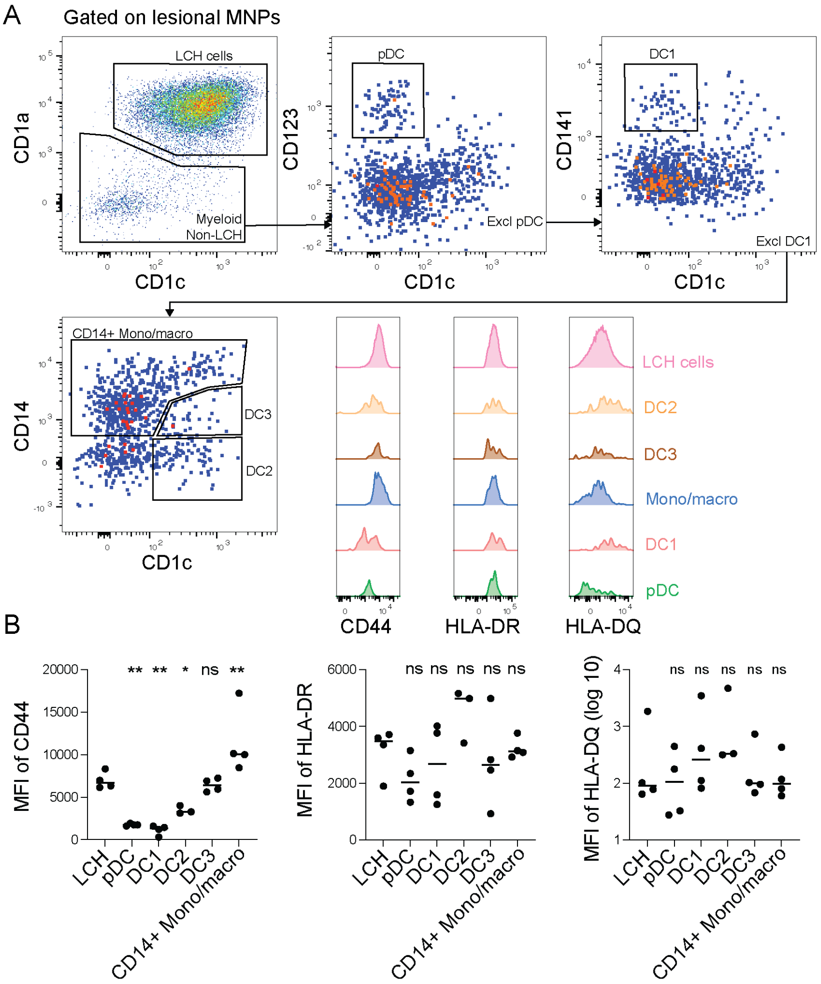

Fig. S14. CD44, HLA-DR, HLA-DQ levels on the whole LCH cell population. (A) Gating strategy and histograms. (B) Quantification, levels compared to the levels detected on LCH cells. ANOVA with Holm-Šídák's multiple comparisons test for multiple comparisons between LCH cells and the remaining subsets, p value : * <0.05, ** <0.01, *** <0.001

Table S1. Patient characteristics

	Patient 1	Patient 2	Patient 3	Patient 4	Patient 5	Patient 6
**General details**						
Sex	Male	Male	Male	Female	Male	Male
Age at LCH diagnosis	6 y 3 mo	33 mo	10 y	19 mo	27 mo	32 mo
Organs involved at diagnosis	Bone	Bone (incl. CNS-risk)	Bone	Bone	Bone	Bone (incl. CNS-risk), skin
Organs involved at sampling point	Bone	Bone	Bone	Bone	Bone	Bone (incl. CNS-risk), skin
CD1a/CD207 positive specimen	Bone	Bone	Bone	Bone	Bone	Bone, skin
Specifications of specimen studied	Bone aspirate	Bone aspirate	Bone curettage	Bone aspirate	Bone curettage	Bone aspirate
Disease status at sampling	Active	Active	Active	Active	Active	Active
MAPK pathway status/mutation<br>- method used<br>- specimen tested	BRAFV600E<br>PCR<br>Bone	BRAFV600E<br>PCR<br>Bone	BRAFV600E<br>PCR<br>Bone	Negative[#]<br>PCR<br>Bone	BRAFV600E<br>PCR<br>Bone	BRAFV600E<br>PCR<br>Bone
Organs involved since diagnosis (until last follow-up)	Bone	Bone	Bone	Bone	Bone (incl. CNS-risk), skin, CNS	Bone, skin, CNS, possibly lungs
Maximal extent of disease	SS	MF	MF	SS	MS RO-	MS RO-
**Laboratory values at sampling**						
-ESR (mm/hour)/CRP (ng/L)	24/-	36/1	19/-	24/-	13/2	34/11
-Hemoglobin (g/L)	140	118	130	112	108	118
-WBC/ANC ($x10^9$/L)	10.5/4.9	9.5/3.6	6.3/3.0	8.0/3.1	6.6/3.4	6.3/4.2
-Platelets ($x10^9$/L)	416	385	199	470	437	437
-Albumin (g/L)	37	37	-	36	38	36
**LCH therapy**						
- ongoing at the sampling	None<br>None	None<br>None	None<br>Pred*, VBL*	None<br>None	None<br>None	None<br>None

- received prior to the sampling<br>- received after the sampling	Methpred[##]	Pred, VBL, 6-MP, MTX, Methpred[##]	Curettage, 6-MP, MTX	Methpred[##]	Curettage, Pred, VCR, Ara-C, 6-MP, MTX	Pred, VBL, Dabrafenib
Comments / other diseases / manifestations			Reactivation 5 y post treatment	Later multiple reactivations	Diabetes Insipidus	Diabetes Insipidus

ANC: absolute neutrophil count; ESR: erythrocyte sedimentation rate, 6-MP: 6-Mercaptopurine; MF: multifocal; MS: Multisystem; MTX: methotrexate; Methpred: Methprednisolone; mo: months; Pred: prednisolone; RO: Risk organ; SS: single system; VBL: Vinblastine; VCR: Vincristine; WBC: white blood count; y: years

[*]Received 5 years prior to the sampling during the initial LCH presentation

[#]Among tested genes: BRAF, MAP2K1, EGFR, KRAS, NRAS, PIK3CA, TP53, PTEN, ALK, ERBB2, ERBB4, FGFR1-3, MET, DDR2, AKT1, SKT11, NOTCH1, CTNNB1, SMAD4, FBXW7

[##]Local steroid injection

Table S2. Materials, antibodies, reagents

**Antibodies for flow cytometry**		
CD1c (clone F10/21A3) BB700	BD	Cat# 746095, RRID: AB_2743468
CD5 (clone UCHT2) BUV395	BD	Cat# 742554, RRID: AB_2740864
CD16 (clone 3G8) BUV737	BD	Cat# 612786, RRID: AB_2833077
CD45 (clone HI30) BUV805	BD	Cat# 564914, RRID: AB_2744401
p53 (clone J159-641.79) AF647	BD	Cat# 560280, RRID: AB_1645430
CD14 (clone M5E2) APC-Cy7	BioLegend	Cat# 301820, RRID: AB_493695
p16 (clone EPR1473) PE	Abcam	Cat# ab209579, RRID: AB_2877634
CCR7 (clone G043H7) BV421	BioLegend	Cat# 353208, RRID: AB_11203894
CD123 (clone 9F5) BV480	BD	Cat# 566133, RRID: AB_2739532
CD45RA (clone HI100) BV510	BioLegend	Cat# 304142, RRID: AB_2561947
CD11b (clone ICRF44) BV570	BioLegend	Cat# 301325, RRID: AB_11150781
FCER1A (clone AER-37) BV605	BD	Cat# 747785, RRID: AB_2872249
CD141 (clone 1A4) BV711	BD	Cat# 563155, RRID: AB_2738033
mTOR (pS2448) (clone O21-404) PE	BD	Cat# 563489, RRID: AB_2736872
CD1a (clone HI149) AF700	BioLegend	Cat# 300120, RRID: AB_528764
CD3 (clone SP34-2) BV650	BD	Cat# 563916, RRID: AB_2738486
CD19 (clone SJ25C1) BV650	BD	Cat# 563226, RRID: AB_2744313
CD20 (clone 2H7) BV650	BD	Cat# 563780, RRID: AB_2744327
HLA-DR (clone L243) BV785	BD	Cat# 307642, RRID: AB_2563461
CD123 (clone 7G3) BUV395	BD	Cat# 564195, RRID: AB_2714171
CD5 (clone UCHT2) BV711	BD	Cat# 563170, RRID: AB_2738044
CD34 (clone 563) BV605	BD	Cat# 745105, RRID: AB_2742712
CD45 (clone HI30) V500	BD	Cat# 560777, RRID: AB_1937324

CD1c (clone L161) BV421	BioLegend	Cat# 331526, RRID: AB_10962909
FCER1A (clone AER-37) PercP	BioLegend	Cat# 334616, RRID: AB_2168079
CD45RA (clone 5H9) FITC	BD	Cat# 556626, RRID: AB_396498
CD88 (clone S5/1) PE/Cy7	BioLegend	Cat# 344308, RRID: AB_11126750
CD206 (clone 19.2) PE/CF594	BD	Cat# 564063, RRID: AB_2732052
CD207 (clone DCGM4) PE	Beckman Coulter	Cat# IM3577, RRID: AB_131233
CD16 (clone 3G8) APC/Cy7	BioLegend	Cat# 302018, RRID: AB_314218
CD141 (clone AD5-14H12) APC	Miltenyi Biotec	Cat# 130-090-907, RRID: AB_244170
CD15 (clone W6D3) BV510	BioLegend	Cat# 323028, RRID: AB_2563400
CD3 (clone UCHT1) BV510	BioLegend	Cat# 300448, RRID: AB_2563468
CD19 (clone HIB19) BV510	BioLegend	Cat# 302242, RRID: AB_2561668
CD7 (clone M-T701) BV510	BD	Cat# 563650, RRID: AB_2713913
CD45 (clone HI30) BV650	BioLegend	Cat# 304044, RRID: AB_2563812
CD74 (clone LN2) APC	BioLegend	Cat# 326811, RRID: AB_2564388
CD44 (clone IM7) FITC	BioLegend	Cat# 103021, RRID: AB_493684
HLA-DP (clone B7/21) PE	BD	Cat# 566825, RRID: AB_2869887
HLA-DQ (clone Tu169) PE-CF594	BD	Cat# 564807, RRID: AB_2738964
CD16 (clone 3G8) PE-CY5	BD	Cat# 555408, RRID: AB_395808
CD123 (clone SSDCLY107D2) PE-Cy5.5	Beckman Coulter	Cat# B20022, RRID: AB_2877635
CD207 (clone MB22-9F5) PE-Vio770	Miltenyi Biotec	Cat# 130-100-586, RRID: AB_2656232
CD83 (clone HB15e) BUV737	BD	Cat# 564441, RRID: AB_2738809
CD115 (clone 9-4D2-1E4) PE-Cy7	BioLegend	Cat# 347308, RRID: AB_2565489
CD1c (clone L161) FITC	BioLegend	Cat# 331517, RRID: AB_2073404
CD276 (clone MIH42) PE-Cy7	BioLegend	Cat# 351008, RRID: AB_2564555
CD59 (clone OV9A2) PE	Thermo Fisher	Cat# 12-0596-41, RRID: AB_10717398

Osteopontin (EPR21139-316) AF488	Abcam	Cat# ab282004
Notch1 (MHN1-519) BB515	BD	Cat# 564781
Notch2 (MHN2-25) BV510	BD	Cat# 742290
Notch3 (MHN3-21) BV605	BD	Cat# 745152
Notch4 (MHN4-2) BV421	BD	Cat# 563905
DLL1 (MHD1-314) BV711	BD	Cat# 745468
JAG1 (W16199B) APC	BioLegend	Cat# 399105
JAG2 (MHJ2-523) PE	BioLegend	Cat# 346904
DLL4 (MHD4-46) BV786	BD	Cat# 744642
**Antibodies for high-content imaging**		
CD207 (clone 929F3.01) unconjugated	Origene	Cat# DDX0362, RRID: AB_1148742
CD134 (OX40) (clone REA621) PE	Miltenyi Biotec	Cat# 130-109-601, RRID: AB_2654932
CD45RO (clone REA611) PE	Miltenyi Biotec	Cat# 130-113-559, RRID: AB_2733819
CLA (clone REA1101) PE	Miltenyi Biotec	Cat# 130-119-043, RRID: AB_2733851
CD20 Cytoplasmic (clone REA543) PE	Miltenyi Biotec	Cat# 130-108-289, RRID: AB_2656084
PDPN (clone REA446) PE	Miltenyi Biotec	Cat# 130-117-687, RRID: AB_2751407
CD44 (clone REA690) PE	Miltenyi Biotec	Cat# 130-113-342, RRID: AB_2726118
CD45RA (clone REA562) PE	Miltenyi Biotec	Cat# 130-113-366, RRID: AB_2726136
CD52 (clone REA164) PE	Miltenyi Biotec	Cat# 130-099-635, RRID: AB_2658645
BATF (clone REA486) PE	Miltenyi Biotec	Cat# 130-107-663, RRID: AB_2651260
CD66b (clone REA306) PE	Miltenyi Biotec	Cat# 130-122-922, RRID: AB_2811406
CD147 (clone REA282) PE	Miltenyi Biotec	Cat# 130-104-490, RRID: AB_2655189
CD88 (C5AR) (clone S5/1) PE	Miltenyi Biotec	Cat# 130-104-285, RRID: AB_2659431
Cytokeratin (clone REA831) PE	Miltenyi Biotec	Cat# 130-112-744, RRID: AB_2651498

Galectin-9 (clone REA435) PE	Miltenyi Biotec	Cat# 130-106-522, RRID: AB_2651800
HLA-DR, DP, DQ (clone REA332) PE	Miltenyi Biotec	Cat# 130-120-715, RRID: AB_2752176
IRF-4 (clone REA201) PE	Miltenyi Biotec	Cat# 130-100-917, RRID: AB_2652515
Syk (clone REA111) PE	Miltenyi Biotec	Cat# 130-099-280, RRID: AB_2653627
CD15 (clone VIMC6) PE	Miltenyi Biotec	Cat# 130-113-485, RRID: AB_2733765
**Samples**		
Cell suspensions from LCH lesions	Department of Pediatric Oncology, Karolinska University Hospital and Division of Pediatric Oncology and Hematology, Skåne University Hospital	N/A
Diagnostic LCH FFPE biopsies	Pediatric Hospital Dr. Juan P. Garrahan, Buenos Aires	N/A
Normal human lymph node, bone marrow and skin FFPE tissues	ProteoGenex	Identifier #:<br>Lymph node 47279A (1),<br>Skin 181144A (1),<br>Bone marrow 80198A (6).
**Assays**		
MACSima™ Imaging System	Miltenyi Biotec	Cat# 130-121-164
Deposited Data		
Smart-seq2 single-cell transcriptome data of lesional cells from pediatric LCH patients	This paper	GEO: GSE173923
10X single-cell transcriptome data of lesional cells from pediatric LCH patients	Halbritter et al., 2019	GEO: GSE133706
Smart-seq2 single-cell transcriptome data of human mononuclear phagocytes	Dutertre et al., 2019	GEO: GSE132566

Bulk RNAseq of human mononuclear phagocytes	Dutertre et al., 2019	GEO: GSE132566
**Software and code**		
Prism 8	Graphpad	https://www.graphpad.com/scientific-software/prism/
FlowJo v.10.5.3	BD	https://www.flowjo.com
SeqGeq	BD	https://www.flowjo.com/solutions/seqgeq
DIVA 6.0	BD	https://www.bdbiosciences.com/en-us
R	The R Foundation	https://www.r-project.org
tSNE	Van der Maaten and Hinton, 2008	https://github.com/jkrijthe/Rtsne/
UMAP	McInnes et al., 2018	https://github.com/lmcinnes/umap/
PhenoGraph	Levine et al., 2015	https://github.com/JinmiaoChenLab/RPhenoGraph/
Monocle 3	Cao et al., 2019	https://cole-trapnell-lab.github.io/monocle3/
Ingenuity Pathway Analysis	QIAGEN	https://www.qiagenbioinformatics.com/products/ingenuity-pathway-analysis/
RSEM	Li and Dewey, 2011	https://deweylab.github.io/RSEM/
Seurat 3.1	Butler et al., 2018	https://github.com/satijalab/seurat/
CMAP	Lamb et al., 2006	https://github.com/pespila/CMAP/
LandSCENT v0.99.3	Teschendorff and Enver, 2017	https://aeteschendorff-lab.github.io/software/LandSCENT/
SCENIC	Aibar et al., 2017	https://github.com/aertslab/SCENIC
CellPhoneDB 2	Efremova et al., 2020	https://github.com/Teichlab/cellphonedb
ImageJ	NIH	https://imagej.nih.gov/ij/
MorphoLibJ	Legland et al., 2016	https://github.com/ijpb/MorphoLibJ
Imaris v8.4.2	Oxford Instruments	https://imaris.oxinst.com
